# Pharmacological Inhibition of Small Extracellular Vesicle Secretion by ALK5i SD‐208 via Lysosomal Rerouting of CD63+ Compartments

**DOI:** 10.1002/jev2.70362

**Published:** 2026-08-29

**Authors:** Rahul Sanwlani, Georgina H. Thompson, Kyle Bramich, Ching‐Seng Ang, Millie L. Trowbridge, Chris E. Duringer, Vlad Stolojan, Sean M. Davidson, Konstantinos Savvatis, John H. McVey, Suresh Mathivanan, Aled Clayton, Patrizia Camelliti

**Affiliations:** ^1^ School of Biosciences University of Surrey Guildford UK; ^2^ Department of Biochemistry La Trobe Institute for Molecular Science La Trobe University Melbourne Victoria Australia; ^3^ The Bio21 Molecular Science and Biotechnology Institute University of Melbourne Parkville Victoria Australia; ^4^ Advanced Technology Institute University of Surrey Guildford UK; ^5^ The Hatter Cardiovascular Institute University College London London UK; ^6^ Inherited Cardiovascular Diseases Unit Barts Heart Centre London UK; ^7^ Institute for Cardiovascular Sciences University College London London UK; ^8^ NIHR University College London Hospitals Biomedical Research Centre University College London London UK; ^9^ Division of Cancer & Genetics School of Medicine Cardiff University Cardiff UK

**Keywords:** ALK5 inhibitor (SD‐208), cardiac fibroblasts, extracellular vesicles, fibrosis, hypertrophic cardiomyopathy, lysosome–autophagy pathway, myofibroblasts, TGF‐β signalling, vesicle trafficking

## Abstract

Pharmacological tools to selectively modulate extracellular vesicle (EV) secretion are scarce. Here, we identify the ALK5 (TGF‐β receptor I) inhibitor SD‐208 as a potent suppressor of small EV (sEV) secretion that acts independently of its canonical anti‐fibrotic activity. SD‐208 not only reversed myofibroblast activation but also markedly inhibited sEV secretion. Strikingly, this inhibitory effect persisted in non‐activated cardiac fibroblasts and non‐fibrotic HEK293 cells, demonstrating that SD‐208 regulates EV secretion through mechanisms uncoupled from TGF‐β/Smad signalling. Mechanistic analyses revealed that SD‐208 disrupts vesicle trafficking rather than EV biogenesis. Reduced secretion of CD63^+^ EVs was accompanied by intracellular accumulation of CD63^+^ structures and their selective diversion into LAMP1^+^ lysosomes. Proteomic profiling of SD‐208‐treated and control HEK293 cells and cardiac fibroblasts revealed dysregulation of vesicle trafficking pathways, enrichment of ubiquitin ligase complexes, and enhanced endosome‐to‐lysosome transport. Together, these findings demonstrate that SD‐208 diverts CD63^+^ multivesicular bodies (MVBs) from a secretory fate toward lysosomal degradation. This work identifies SD‐208 as a small‐molecule tool to interrogate the secretory‐versus‐degradative fate of MVBs and uncovers a new regulatory link between lysosomal pathways and EV trafficking. Beyond its established role as an anti‐fibrotic agent, SD‐208 provides mechanistic and therapeutic opportunities for the control of EV secretion in diseases such as fibrosis, cardiac remodelling, hypertrophic cardiomyopathy, and cancer.

## 1 Introduction

Existing tools to modulate EV secretion are available; however, most lack specificity and act indirectly, complicating mechanistic interpretation of their effects on vesicle biogenesis, trafficking, or release (Quadri et al. 2022; Catalano and O'Driscoll 2020). Current strategies rely largely on genetic manipulation of ESCRT components or Rab GTPases, or indirect modulation via upstream signalling pathways, approaches that complicate mechanistic interpretation because altered EV release may reflect secondary effects on cell activation or viability rather than specific interference with vesicle biogenesis, trafficking or exocytic release. Small molecules such as GW4869, manumycin A, and calpeptin often impair cellular function or act through poorly defined mechanisms (Catalano and O'Driscoll 2020; Tricarico et al. 2017; Hyenne et al. 2015; Kim et al. 2022; Mimi and Hasan 2025; Rezaie et al. 2022; van Niel et al. 2018). Identifying compounds that selectively redirect multivesicular bodies (MVBs) between secretion and degradation without disrupting cell health represents a critical unmet need.

Extracellular vesicles are increasingly recognised as central mediators of intercellular communication in cardiovascular and fibrotic diseases (Rai et al. 2024). In the heart, activated fibroblasts release EVs that promote cardiomyocyte hypertrophy, extracellular matrix remodelling, inflammatory signalling, and maladaptive tissue remodelling. In hypertrophic cardiomyopathy (HCM) and related fibrotic conditions, altered EV secretion contributes to propagation of pathological signalling between cardiac cell types (Sanwlani and Camelliti 2026). Therefore, pharmacological modulation of EV release represents a potentially powerful strategy to interrupt disease‐driving intercellular communication networks.

SD‐208, a selective inhibitor of TGF‐β receptor I (ALK5), is well‐characterised for its anti‐fibrotic activity in oncology and cardiovascular disease, where it blocks Smad3 phosphorylation and extracellular matrix deposition (Sanwlani et al. 2024; Tan et al. 2010; Uhl et al. 2004; Frangogiannis 2012; Driesen et al. 2014; Mohammad et al. 2011). However, its potential effects on EV secretion have not been explored. Given emerging evidence that endosomal trafficking intersects with both fibrotic signalling and vesicle release, and that ALK5 inhibitors may influence kinases regulating membrane dynamics, we hypothesised that SD‐208 might modulate EV secretion independently of its canonical TGF‐β/Smad pathway effects (Wang et al. 2022; Siegert et al. 2018; He et al. 2015; Miller et al. 2018; Shelke et al. 2019).

To dissect these possibilities, we employed three complementary cellular systems representing decreasing levels of fibrotic signalling: (i) primary cardiac fibroblasts from HCM patients as highly activated myofibroblasts with robust TGF‐β/ALK5 activity; (ii) non‐activated, TGF‐β‐responsive cardiac fibroblasts; and (iii) non‐fibrotic HEK293 cells with minimal endogenous TGF‐β activity. This framework allowed us to distinguish EV‐regulatory effects from anti‐fibrotic actions. These cardiac fibroblast models provide translational relevance, as fibroblast‐derived EVs promote cardiomyocyte hypertrophy and adverse remodelling in HCM and other cardiac pathologies (Thompson et al. 2024; Sanwlani et al. 2025; Sanwlani et al. 2025; Lyu et al. 2015; Tian et al. 2020; Ranjan et al. 2021; Bang et al. 2014).

Here, we identify SD‐208 as a pharmacological suppressor of small EV (sEV) secretion that acts independently of its canonical anti‐fibrotic signalling effects. SD‐208 markedly reduced sEV release across all models without affecting cell viability, proliferation, or metabolism. Mechanistically, it disrupted vesicle trafficking rather than biogenesis, diverting CD63^+^ MVBs from plasma membrane fusion toward lysosomal degradation through a pathway distinct from canonical autophagy. These findings establish SD‐208 as a novel small‐molecule tool to interrogate MVB fate and provide proof‐of‐concept that EV secretion can be selectively modulated at the level of endosomal trafficking, with potential therapeutic implications for EV‐driven diseases including cardiac remodelling, HCM, and cancer (Lyu et al. 2015; Tian et al. 2020; Ranjan et al. 2021; Bang et al. 2014; Costa‐Silva et al. 2015; Lucotti et al. 2025; Fonseka et al. 2019).

## 2 Methods

### 2.1 SD‐208 and TGF‐β Treatments

SD‐208 (S7071, Sigma–Aldrich) was dissolved in DMSO to make a 6 mM stock solution which was aliquoted and stored at −20°C until required. To treat cells, the stock solutions were diluted in culture medium to obtain desired concentrations and used to incubate cells for required time at 37°C with 5% CO_2_ in serum starved conditions. Recombinant human TGF‐β1 (PHP143B, Bio‐Rad) was reconstituted in sterile, culture grade water to make a stock solution at 0.1 mg/mL which was aliquoted and stored at −20°C until required. Prior to treatment with TGF‐β1, cells were serum starved overnight followed by treatment with desired concentration and duration. During TGF‐β1 treatment cells were supplemented with bovine serum albumin (BSA) (Sigma–Aldrich) solution (0.1% w/v).

### 2.2 Human Primary Cardiac Fibroblast Isolation

Primary adult human cardiac fibroblasts were isolated from HCM patient septal myectomies (Barts Heart Centre, ethics IRAS185164) (Figure S1). Myectomies were collected in cold sterile Dulbecco's phosphate‐buffered saline (DPBS) supplemented with 1% penicillin/streptomycin (P/S, Gibco, 15070‐063), washed six times in fresh sterile DPBS containing 1% P/S, and cut into small pieces (∼4 mm^3^) with sterile scissors. After removal of DPBS, tissue pieces were incubated in 0.25% trypsin‐EDTA for 3 min, followed by trypsin inactivation with the same volume of complete fibroblast medium (Dulbecco's Modified Eagle Medium (DMEM), 15% (v/v) foetal calf serum (FCS, Labtech), 1% GlutMAX (Thermo Fisher), 1% P/S). The tissue pieces were cut into smaller pieces (<1 mm^3^) and plated onto petri dishes (gridded dishes, 60 mm) pre‐coated with fibronectin (Sigma, F2006, 5 µg/mL fibronectin in DPBS coated for 4 h at 37°C). Tissue pieces were plated onto petri dishes with a 5 mm distance in 0.5 mL of fibroblast medium and incubated at 37°C for 2–3 h before addition of further 1 mL of medium. Explants were incubated with regular medium changes (every 48–72 h) and cardiac fibroblasts allowed to grow until dishes were >80% confluent. Cells were detached using gentle trypsinisation (0.25% trypsin) and transferred to culture flasks. Once the flasks were confluent, cells were lifted using Accutase (Sigma, A6964) and cryopreserved (10% DMSO in FBS; passage 1) in liquid nitrogen until further use. Patient information for HCM tissues has been provided in Table S1.

### 2.3 Cell Culture

HCM primary human cardiac fibroblasts were cultured in complete fibroblast medium. Immortalised cardiac fibroblast (ICF, ventricular, Innoprot) were cultured in FM‐2 medium (ScienceCell) supplemented with 1% FGS‐2 (v/v), 1% P/S (v/v) and 5% FCS. ICFs were cryopreserved in FreezeMe Two freezing media (Capricorn scientific, FM2‐F). HEK293 cells (ATCC CRL‐1573) were cultured in DMEM supplemented with 10% (v/v) FCS, 1% P/S (v/v), 1% L‐glutamine and 1% non‐essential amino acids. Primary human cardiac fibroblasts (PromoCell, C‐12375) were cultured using Fibroblast Medium 3 (FM‐3; PromoCell, C‐23025) supplemented with the corresponding supplement mix (PromoCell, C‐39345) according to the manufacturer's instructions. Cells were maintained in the absence of penicillin/streptomycin. Low‐passage cells were cryopreserved in PromoCell cryopreservation medium (C‐29910) and used up to passage 6 for all experiments. Donor details for PromoCell primary human cardiac fibroblasts are provided in Table S1. All cells were maintained in an incubator with 5% CO_2_ at 37°C.

### 2.4 sEV Isolation

Upon incubation or treatment endpoint, conditioned media was collected and processed to isolate sEVs using ultrafiltration and size exclusion chromatography combination. Following collection, conditioned media was centrifuged at 400 × *g* for 6 min. The supernatant was transferred to a fresh tube and the pellet discarded. Next, the supernatant was centrifuged at 500 × *g* for 6 min to pellet the cells. The conditioned media was then centrifuged at 2000 × *g*, 4°C for 20 min. Following successive centrifugations, conditioned media was filtered using 0.22 µm bottle filter. Next, conditioned media was subjected to ultrafiltration using Amicon (UFC901024) 10 kDa molecular weight cutoff filters, centrifuged at 4000 × *g*, 4°C until it was concentrated to 500 µL volume. sEV was isolated using size exclusion chromatography (SEC) with Izon columns (ICO‐35 qEV original/35 nm Gen 2 column). The columns were equilibrated using DPBS, then 500 µL of EV concentrate collected with ultrafiltration was loaded to the top of the column. The elute was collected in a 1.5 mL microcentrifuge tube labelled Fraction 1. Next 500 µL of PBS was added and this was collected in a microcentrifuge tube labelled Fraction 2. This was repeated until 24 fractions were collected. Collected fractions were stored at −80°C. sEVs were enriched in fractions 7–9 which were pooled for further characterisation and analysis.

### 2.5 Nanoparticle Tracking Analysis

The size distribution and abundance of the sEVs collected were measured using nanoparticle tracking analysis (NTA) using a NanoSight LM10‐HS microscope (Malvern Instruments, Malvern, UK). The samples were monitored with the laser Monochromatic laser beam at 405 nm. The NanoSight laser module was washed three times with particle free water to remove background particulate. All parameters and settings were maintained constant throughout the experiment. On the NanoSight programme (NTA version 3.2) the camera level was set to 15, the detection threshold set to 5 and the syringe flow was set to 20. For each sample, 3 videos (90 s each) were captured and analysed for each sample (replicate). Results obtained were analysed with NTA 3.1 (Build 3.1.54) software.

### 2.6 Transmission Electron Microscopy

sEV samples (0.2 mg/mL) were subjected to TEM analysis with Talos 200i transmission electron microscope (Thermo Fisher Scientific) as described previously (Samuel et al. 2021, 2023; Sanwlani et al. 2023). Briefly, samples were applied to mesh carbon‐layered copper grids (Formvar on 3 mm 300 mesh Ni grids, Agar Scientific) and allowed to adsorb for 2 min. Excess sample was removed using blotting paper. Prior to imaging, samples were stained with 10 µL of 2% (w/v) uranyl acetate substrate solution (HAZ UA‐Zero EV Stain, Agar Scientific).

### 2.7 sEV Protein Quantification

To determine the protein content of the sEV containing fractions following size exclusion chromatography, a microBCA assay (Bicinchoninic acid, Thermofisher, 23235) was performed. sEVs (30 µL) were lysed in equal volume of SDS‐lysis buffer (1% (v/v) SDS, 10 mM Tris pH 7.4 in ddH2O). This was diluted in DPBS as necessary to fit the standard curve (on average 1:10) followed by microBCA according to manufacturer's instructions. Absorbance was measured at 562 nm using a plate reader (SpectraMax iD3), and protein concentration was determined by comparison to a BSA standard curve.

### 2.8 sEV Immunophenotyping

sEVs were characterised by immunophenotyping using an immunophenotyping assay similar to an ELISA to detect classical EV surface markers as previously defined (Ní Bhaoighill et al. 2023; Shephard et al. 2021). Briefly, 1 µg of sEVs was added per well to a 96‐well high‐protein binding plate (Greiner Bio‐One, #756071) and incubated overnight at 4°C to allow passive adsorption. The following day, wells were blocked with 300 µL of 2% BSA (R&D Systems, #841380) for 2 h at RT on a plate shaker, followed by washing with 1× wash buffer (Uniogen, 42‐01) using an automated plate washer. Primary antibodies were diluted in 1% BSA in DPBS and added at 100 µL/well, then incubated for 1 h at RT on a plate shaker. The antibodies used were:

anti‐CD63 (3 µg/mL; Bio‐Rad, MCA2142)
anti‐CD81 (2 µg/mL; Bio‐Rad, MCA1847EL)
anti‐CD9 (2 µg/mL; R&D Systems)
anti‐Calnexin (2 µg/mL; R&D Systems, MAB1880)
mouse IgG isotype control (1 µg/mL; Santa Cruz, sc‐3879)

Following incubation, wells were washed, and biotinylated goat anti‐mouse IgG secondary antibody (PerkinElmer, #823001EA) diluted 1:2500 in 1% BSA/DPBS was added (100 µL/well) for 1 h at RT with shaking. After washing, streptavidin‐europium conjugate (PerkinElmer, #1244‐360) was diluted 1:1000 in Red Assay Buffer (Kaivogen, #42‐02), added at 100 µL/well, and incubated for 45 min at RT. Plates were washed extensively (six times), and 100 µL of europium intensifier (Kaivogen, #KG1212) was added per well for 5 min at RT. Time‐resolved fluorescence was measured using a BMG PHERAstar plate reader to quantify marker expression.

### 2.9 Flow Cytometry

Flow cytometry analysis was performed to further characterise the cells. Cells were cultured to confluency under appropriate conditions, followed by harvesting using Accutase. Briefly, cells were washed with 3 mL DPBS, then incubated with 3 mL Accutase (room temperature, 10 min) until cells adopted a rounded morphology. Cells were gently detached by tapping the flask and collected by centrifugation at 500 × g for 5 min. The cell pellet was washed in DPBS and resuspended at 100 µL per sample, depending on the number of conditions to be tested. All subsequent steps were performed at 4°C. Resuspended cells (100 µL) were aliquoted into 1.5 mL microcentrifuge tubes. For viability staining, one sample was incubated with Zombie Red fixable viability dye (1:100; BioLegend, 423109) for 30 min in the dark. For all other samples, 500 µL of Cell Staining Buffer (BioLegend, 420201) was added, followed by centrifugation at 400 × g for 5 min. Pellets were resuspended in 100 µL Cell Staining Buffer.

For intracellular staining, samples were fixed in 500 µL Fixation Buffer (BioLegend, 420801) for 20 min, then centrifuged at 400 × g for 5 min. These samples were subsequently washed and permeabilised using 100 µL of 1× Intracellular Permeabilization (IP) Buffer (BioLegend, 421002), followed by a second centrifugation step (400 × g, 5 min). This wash‐permeabilisation cycle was repeated once. Primary antibodies (Table S2) were added to each sample and incubated for 30 min in the dark. Samples were then washed twice using Cell Staining Buffer (for surface staining) or IP Buffer (for intracellular staining). For unconjugated primary antibodies, a fluorophore‐conjugated secondary antibody (Table S2) was added and incubated for 30 min, followed by two additional washes. All samples were resuspended in 500 µL Cell Staining Buffer, transferred into FACS tubes, wrapped in foil, and stored on ice until acquisition. Flow cytometry was performed using a BD Celesta flow cytometer (BD Biosciences). Detector voltages were kept consistent across all samples to ensure comparability between fluorophores.

### 2.10 Immunofluorescence

Cells were cultured on glass‐bottomed chamber slides under appropriate conditions. Following treatment, cells were washed twice with DPBS and fixed with 1% paraformaldehyde (PFA; Alfa Aesar, 43368) for 15 min at room temperature. Cells were then washed five times with DPBS on a shaker (5 min per wash). Permeabilisation was performed using 0.1% Triton X‐100 in DPBS for 10 min. Cells were subsequently blocked in a solution containing 1% BSA and 0.1% Triton X‐100 in DPBS for 1 h at room temperature. After blocking, primary antibodies (Table S2) were added and incubated overnight at 4°C. The following day, cells were washed five times with DPBS (5 min per wash on a shaker), followed by incubation with appropriate fluorophore‐conjugated secondary antibodies (Table S2) for 2 h in the dark at RT. A well without primary antibody but with secondary antibody was included as a negative control. After secondary antibody incubation, samples were washed five times again with DPBS. Cells were counterstained with DAPI (4′,6‐diamidino‐2‐phenylindole) for 5 min, followed by five additional DPBS washes. The plastic chamber walls were carefully removed, and the coverslips were mounted using Fluoromount‐G mounting medium (Invitrogen, 00‐4958‐02). Imaging was performed and processed using Nikon Eclipse TS2 fluorescence microscope and NIS Elements BR software (version 5.01, Nikon). For confocal microscopy, Nikon A1M confocal microscope and DS‐Q1 widefield camera (on an Eclipse TiE microscope) and Nikon NIS‐Elements platform were used.

### 2.11 Whole Cell Lysate Preparation and Western Blotting

Cells were lysed using SDS lysis buffer immediately following treatment. The appropriate volume of lysis buffer was added directly onto cells and evenly spread using a cell lifter/scraper (TrueLine) on ice. Whole cell lysates were collected into 1.5 mL microcentrifuge tubes and briefly sonicated to shear nucleic acids. Protein concentrations were quantified using the Bicinchoninic Acid (BCA) Protein Assay Kit (Pierce BCA, Thermo Fisher Scientific), and lysates were stored at −80°C until further analysis.

For SDS‐PAGE, equal amounts of protein were adjusted to a final volume of 13 µL and mixed with 5 µL of 4× LDS sample buffer (Invitrogen, NP0007) and 2 µL of 10× 200 mM dithiothreitol (DTT; Invitrogen, NP0004). Samples were denatured and reduced by heating at 70°C for 10 min. Protein samples and 5 µL of protein ladder (Thermo Fisher, 26619) were loaded onto commercial SDS‐PAGE gels (Invitrogen, NP0321) and run in 1× MES SDS running buffer (Invitrogen, NP002) at 165 V for 30–60 min. Proteins were transferred onto nitrocellulose membranes (Thermo Scientific) using a semi‐dry transfer system (Bio‐Rad, Trans‐Blot SD) with 1× transfer buffer (Invitrogen, NP006‐1). Prior to transfer, the membrane was equilibrated in the same buffer. Transfer was conducted at 15 V for 30 min. Membranes were blocked in 10% (w/v) skim milk prepared in TTBS buffer (100 mM Tris‐HCl, pH 7.5, 150 mM NaCl, 0.05% Tween‐20) for 45 min at room temperature. Following three 10‐minute washes in TTBS, membranes were incubated overnight at 4°C with the appropriate primary antibody (Table S3). After primary antibody incubation, membranes were washed three times in TTBS (10 min each) and then incubated with the corresponding IRDye‐conjugated secondary antibody (LI‐COR) (Table S4) for 1 h at room temperature. Final washes (3 × 10 min) were performed in TTBS before imaging. Blots were visualized using the Odyssey CLx Imaging System (LI‐COR), and band intensities were quantified using ImageJ (version 1.54). Equal amounts of protein were loaded per lane based on BCA quantification, and densitometric values were normalised to GAPDH as a loading control.

### 2.12 Trypan Blue Exclusion Viability Assay

Cells (1 × 10^5^) were initially seeded in a 6‐well plate in 2 mL of culture medium and incubated at 37°C with 5% CO_2_. The cells were trypsinised using 0.25% (v/v) Trypsin‐EDTA (Gibco) or harvested with Accutase (Sigma), and collected by centrifugation at 1500 × *g* for 5 min. The supernatant was removed, and the pellet was resuspended in 15 mL of culture medium. Aliquot of culture medium containing cells was mixed with 0.4% (v/v) Trypan Blue stain (Gibco) with 1:1 ratio and loaded onto cell Countess chamber and inserted into the cell Countess automated cell counter II FL (Invitrogen) to analyse cell number and viability.

### 2.13 MTS Assay

Cell viability and metabolic activity were assessed using the MTS assay (3‐(4,5‐dimethylthiazol‐2‐yl)‐5‐(3‐carboxymethoxyphenyl)‐2‐(4‐sulfophenyl)‐2H‐tetrazolium, inner salt), with the CellTiter 96 AQueous One Solution Cell Proliferation Assay kit (Promega, G3582). Cells were seeded in clear, flat‐bottom 96‐well plates at a density of 5000 cells per well. Following overnight incubation cells were treated for desired timepoints. Following treatment, media was aspirated carefully without disrupting the cells and replaced with 100 µL of fresh media. Twenty microliters of CellTiter 96 AQueous One Solution reagent was added to the media. Plates were wrapped in aluminium foil loosely to protect from light and incubated at 37°C with 5% CO_2_ for 2 h. Absorbance was measured using a SpectraMax iD3 plate reader at 490 and 630 nm. Background absorbance at 630 nm was subtracted from the 490 nm values to yield corrected absorbance representing metabolic activity.

### 2.14 Collagen Secretion Assay

To determine and quantify extracellular collagen secretion by cardiac fibroblasts, cells were cultured and at culture/treatment endpoint conditioned media was collected. Conditioned media was processed by centrifugation at 500 × *g* for 5 min followed by 2000 × *g* for 15 min to remove cells and debris. The processed media was then filtered with 0.22 µm syringe filters. Extracellular, secreted collagen in the conditioned media was quantified with Sirius Red total collagen detection assay plate kit (Chondrex, inc., 9062P) following manufacturer's instructions.

### 2.15 RNA Extraction, cDNA Synthesis and qRT‐PCR

#### 2.15.1 Total RNA for CF Characterisation

Total RNA was isolated using the RNeasy RNaqueous‐4‐PCR kit (Thermo Fisher Scientific, AM1914). Cells were washed once with DPBS, harvested with Accutase, collected and centrifuged at 500 × *g* for 5 min. Pellets were resuspended in 300 µL lysis/binding solution and an equal volume of 64% ethanol was added. After brief vortexing, the mixture was loaded onto a spin column in a collection tube and centrifuged at 10,000 g for 1 min. The flow‐through was discarded and the column was washed with 700 µL Wash Buffer 1 (10,000 × *g*, 1 min), followed by two washes with 500 µL Wash Buffer 2/3 (10,000 × *g*, 30 s each). The column was dried using centrifugation at 10,000 × *g* for 30 s, transferred to a fresh collection tube, and RNA was eluted by applying 50 µL of pre‐warmed (80°C) Elution Solution to the membrane (10,000 × *g*, 30 s), followed by a second elution with 20 µL. Eluates were stored at −80°C until use.

#### 2.15.2 cDNA Synthesis

cDNA was synthesised from total RNA using the SuperScript VILO cDNA Synthesis Kit (Invitrogen, 11756050) according to the manufacturer's instructions. For each reaction, 14 µL RNA was combined with 4 µL VILO Reaction Mix and 2 µL Enzyme Mix, gently vortexed, briefly centrifuged, and incubated in a thermocycler as per kit protocol. Resulting cDNA was diluted with RNase‐free water as required for downstream quantitative PCR.

#### 2.15.3 Real‐Time Quantitative PCR (RT‐qPCR)

Gene expression was quantified using TaqMan chemistry on a QuantStudio 5 Real‐Time PCR System (Thermo Fisher Scientific) under standard cycling conditions. For each reaction, a 20 µL final volume contained 10 µL Absolute QPCR Master Mix (Thermo Fisher Scientific, AB1139A), 1 µL TaqMan Gene Expression Assay (20×), 4 µL nuclease‐free water, and 5 µL cDNA. A master mix was prepared as: (10 µL Master Mix + 1 µL Assay + 4 µL water) × *N*, where *N* = (number of samples × 2 technical replicates) + 3 (excess). Fifteen microlitres of master mix were dispensed per well of a 96‐well plate and 5 µL cDNA added. Each biological condition was run in triplicate, with technical duplicates per sample.

The following TaqMan assays were used: *B2M* (endogenous control, Hs00984230_m1), *ACTA2* (Hs00426835_g1), *COL1A1* (Hs00164004_m1), *COL3A1* (Hs00943809_m1), *FAP* (Hs00990791_m1), *POSTN* (Hs01566750_m1), *IL6* (Hs00174131_m1), and *IL11* (Hs01055413_g1).

Quantification cycle (Ct) values were exported and analysed using the ΔΔCt method with *B2M* as the endogenous control; data are reported as relative expression (fold change vs. the appropriate calibrator). Where indicated, expression was additionally summarised as 2^−ΔCt^.

### 2.16 Proteomic Sample Preparation With In‐Solution Protein Digestion

Protein digestion for proteomic analysis was performed as previously defined by us using S‐Trap micro spin columns (Protifi) according to the manufacturer's protocol, as previously described (Sanwlani et al. 2023). Briefly, 30 µg of total protein was brought to a final volume of 23 µL using SDS‐based lysis buffer. Reduction was performed by adding 1 µL of 120 mM tris(2‐carboxyethyl)phosphine (TCEP; Sigma–Aldrich) in 50 mM ammonium bicarbonate, followed by incubation at 55°C for 15 min. Alkylation was then carried out by adding 1 µL of 500 mM iodoacetamide (Sigma–Aldrich) and incubating the samples in the dark at room temperature for 10 min. To acidify the samples, 2.5 µL of phosphoric acid (27.5% in ddH_2_O) was added, followed by brief vortexing. Protein precipitation and binding were facilitated by adding 165 µL of S‐Trap binding buffer (prepared by mixing 1 mL of 1 M Tris‐HCl with 9 mL molecular‐grade methanol) to the acidified lysate. Samples were loaded onto S‐Trap columns and centrifuged at 4000 × *g* for 30 s. The columns were then washed three times with 150 µL of binding buffer, each followed by centrifugation at 4000 × *g* for 30 s. Proteins were digested on‐column overnight at 37°C using sequencing‐grade modified trypsin (Promega) at a 1:10 (enzyme: protein) ratio. Sequential peptide elution was carried out using three elution buffers added consecutively: 40 µL of 50 mM ammonium bicarbonate (Elution Buffer 1), 40 µL of 0.2% formic acid (Elution Buffer 2), and 40 µL of 50% acetonitrile in ddH_2_O (Elution Buffer 3). Each elution step was followed by centrifugation at 4000 × *g* for 1 min. Eluted peptides were pooled, freeze‐dried, and reconstituted in 2% acetonitrile containing 0.05% trifluoroacetic acid (TFA) prior to LC‐MS/MS analysis.

### 2.17 LC‐MS/MS

LC‐MS/MS analysis was performed using an Orbitrap Exploris 480 mass spectrometer (Thermo Fisher Scientific) as previously defined (Samuel et al. 2023; Fonseka et al. 2021). The LC system was equipped with an Acclaim PepMap nano‐trap column (Dionex‐C18, 100 Å, 75 µm × 2 cm) and an Acclaim PepMap RSLC analytical column (Dionex‐C18, 100 Å, 75 µm × 50 cm). Tryptic peptides were injected into the enrichment column at an isocratic flow of 5 µL/min of 2% (v/v) acetonitrile containing 0.1% (v/v) formic acid for 6 min, before the enrichment column was switched in‐line with the analytical column. The mobile phases consisted of 0.1% (v/v) formic acid in water (solvent A) and 100% (v/v) acetonitrile in 0.1% (v/v) formic acid (solvent B). The gradient elution profile was as follows: (i) 0–6 min at 3% B; (ii) 6–35 min, 3%–22% B; (iii) 35–40 min, 22%–40% B; (iv) 40–45 min, 40%–80% B; (v) 45–50 min, 80% B; (vi) 50–51 min, 80%–3% B, followed by equilibration at 3% B for 10 min before the next sample injection.

The mass spectrometer was operated in data‐dependent acquisition (DDA) mode with full MS1 spectra acquired in positive ionization mode at 120,000 resolution (Tune version 2.0.182.25). The ‘top speed’ acquisition mode was employed with a 3‐s cycle time, targeting the most intense precursor ions with charge states between +2 and +7. MS/MS analyses were performed using 1.2 *m*/*z* isolation window with the quadrupole, followed by higher‐energy collisional dissociation (HCD) fragmentation with normalized collision energy of 30%. MS2 spectra were acquired at 15,000 resolution with dynamic exclusion activated for 20 s. The automatic gain control (AGC) target was set to standard with automatic maximum injection time.

### 2.18 Database Search and Protein Identification

Protein identification and quantification were performed using MaxQuant proteomics software package (version 2.0.1.0). MS/MS spectra were searched against the reviewed UniProt human proteome database (Homo sapiens; taxonomy ID 9606). Default instrument‐specific search parameters were applied with the following specifications: trypsin was set as the cleavage enzyme with a maximum of two missed cleavages allowed. Carbamidomethylation of cysteine was specified as a fixed modification, while oxidation of methionine and acetylation of protein N‐termini were set as variable modifications. Both protein and peptide identifications were filtered to a maximum false discovery rate (FDR) of 1% using a target‐decoy approach. The ‘match between runs’ feature was enabled to improve peptide identification across samples. Protein quantification was performed using intensity‐based absolute quantification (iBAQ) values, which provide a measure of absolute protein abundance by dividing protein intensity by the number of theoretically observable peptides for each protein.

### 2.19 Bioinformatics and Statistical Analysis

Proteomic analysis to generate Venn diagrams, heatmaps and gene enrichment analysis plots was performed using the FunRich software (Fonseka et al. 2021) and Enrichr tool (Evangelista et al. 2023). Principal component analysis was performed using MetaboAnalyst tool (Version 6.0) (Pang et al. 2024). Volcano plots and dot plots were generated using GraphPad Prism 10.2.3 software. Statistical significance was determined with student's t‐test and one way analysis of variance (ANOVA) with the Turkey‐Kramer multiple comparison post‐hoc test using GraphPad Prism 10.2.3 software.

## 3 Results

### 3.1 Isolation and Characterisation of Primary Human Cardiac Fibroblasts From HCM Patients

SD‐208, a selective inhibitor of TGF‐β receptor I (ALK5), has been extensively studied for its anti‐fibrotic properties in various organ systems, including cardiac tissue (Sanwlani et al. 2024; Uhl et al. 2004; Driesen et al. 2014; Mohammad et al. 2011) but not in HCM. In HCM, cardiac fibroblasts differentiate into activated myofibroblasts, contributing significantly to pathological fibrosis and disease progression (Bageghni et al. 2018; Fujiu and Nagai 2014; Pedrotty et al. 2009). To investigate the effects of SD‐208 on primary human cardiac fibroblasts in the context of HCM, we established a protocol for isolating and culturing these cells directly from patient tissue.

Primary human cardiac fibroblasts were isolated from septal myectomy specimens obtained from HCM patients undergoing corrective cardiac surgery (Figure S1). The isolation protocol involved enzymatic digestion of tissue fragments followed by explant culture, allowing cardiac fibroblasts to migrate and proliferate from the tissue pieces over a period of 10–14 days. As shown in Figure S1, confluent fibroblast cultures were successfully established and subsequently expanded for experimental use. All downstream experiments were performed on isolated and cultured cells; no analyses were conducted directly on tissue samples.

To confirm the identity and activation status of the isolated cells, comprehensive characterisation was performed using multiple complementary approaches (Figure S2). Immunofluorescence analysis revealed robust expression of key fibroblast markers, including α‐smooth muscle actin (α‐SMA) and fibronectin, distributed throughout the cell populations (Figure S2A). The cytoskeletal protein vimentin was also abundantly expressed, confirming the mesenchymal origin of the isolated cells. Notably, the expression pattern of α‐SMA, characterised by organised stress fibre formation, indicated that these cells had adopted an activated myofibroblast phenotype consistent with their pathological tissue origin.

To further validate cell identity and assess culture composition, flow cytometric analysis was performed (Figure S2B). The isolated cardiac fibroblasts demonstrated high expression of CD90 (Thy‐1), with >80% of cells positive, supporting enrichment for a fibroblast population. In contrast, contamination with other cardiac cell types was minimal, as evidenced by negligible expression of CD45 (immune cell marker, <0.3%) and CD31 (endothelial cell marker, <0.03%). Approximately 60% of cells were α‐SMA‐positive, consistent with a substantial proportion of activated myofibroblasts.

Importantly, α‐SMA was used here as a marker of activation rather than fibroblast identity. The presence of an α‐SMA‐negative fraction is therefore expected and likely reflects phenotypic heterogeneity within the fibroblast population, including less activated cells. Taken together, these data indicate that the cultures represent a highly enriched cardiac fibroblast population with heterogeneous activation states, rather than a mixture of distinct cell lineages.

Finally, to characterise the activation state of the isolated fibroblasts at the molecular level, quantitative RT‐PCR analysis was performed to assess expression of established myofibroblast markers (Figure S2C). The cells demonstrated expression of multiple fibroblast activation markers plotted relative to the housekeeping gene *B2M*, including *ACTA2* (encoding α‐SMA), collagen genes *COL1A1* and *COL3A1*, periostin (*POSTN*), fibroblast activation protein (*FAP*), and pro‐inflammatory cytokines *IL‐6* and *IL‐11* (Frangogiannis 2021; Kong et al. 2014; Schafer et al. 2017). This gene expression profile indicated that the isolated cells exhibited a highly activated myofibroblast phenotype characteristic of pathological cardiac remodelling.

Collectively, these comprehensive characterisation studies confirmed the successful isolation and culture of primary human cardiac myofibroblasts from HCM patient tissue. The cells demonstrated the expected morphological, phenotypic, and molecular characteristics of activated cardiac fibroblasts, providing an appropriate cellular model to investigate the anti‐fibrotic effects of SD‐208 in the context of human cardiac pathology.

### 3.2 SD‐208 Attenuates Myofibroblast Activation in Primary Human Cardiac Fibroblasts

**FIGURE 1:**
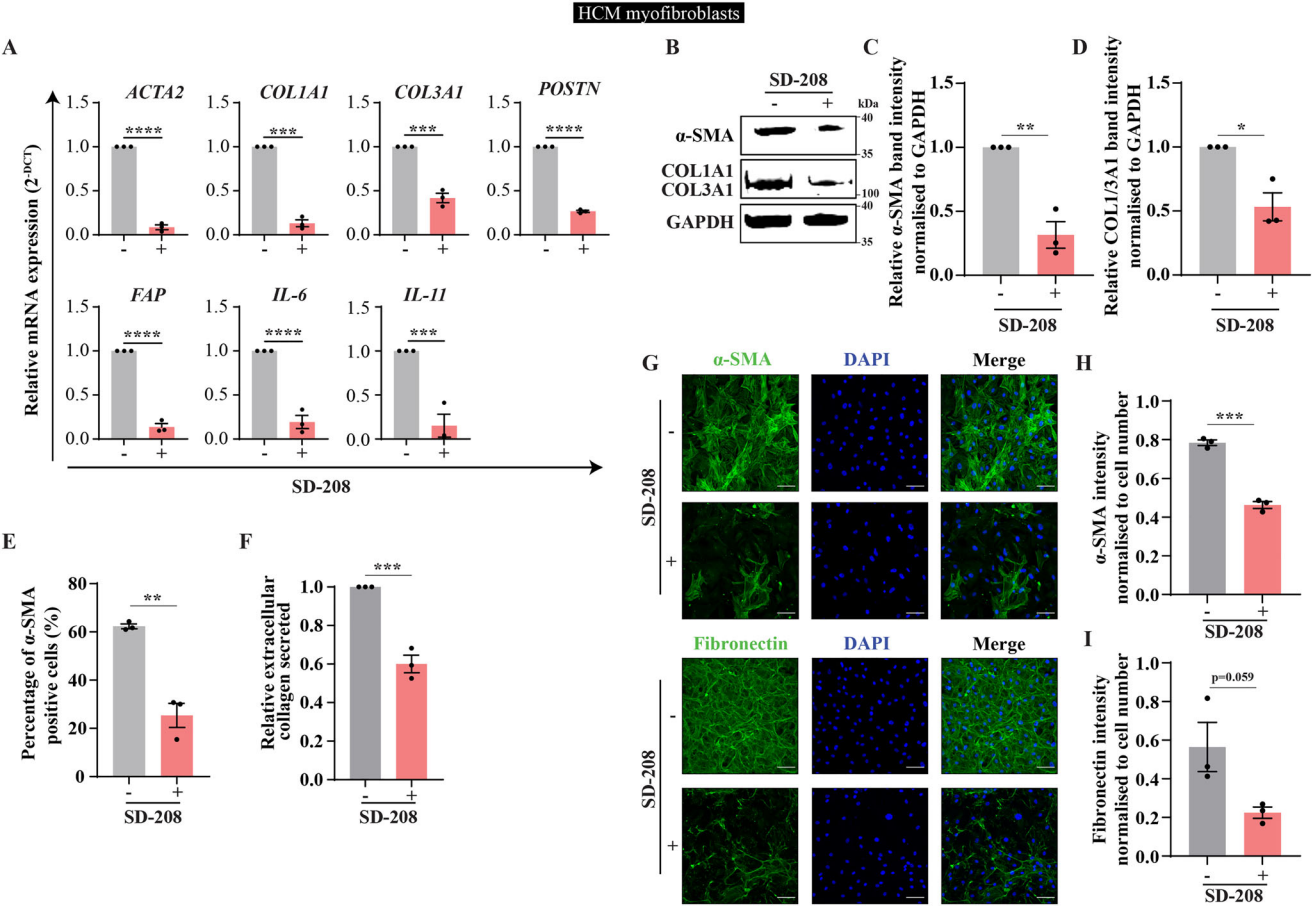
SD‐208 treatment attenuates myofibroblast activation in primary human cardiac fibroblasts. (A) qRT‐PCR analysis of fibroblast activation markers in primary cardiac fibroblasts treated with SD‐208 (3 µM) or vehicle control for 96 h. Expression levels are normalized to *B2M* and shown relative to vehicle control. (B) Representative western blot analysis of α‐SMA and COL1A1 protein expression in cardiac fibroblasts treated with SD‐208 or vehicle control. GAPDH served as loading control. (C, D) Densitometric quantification of α‐SMA (C) and COL1A1 (D) protein levels normalized to GAPDH. (E) Flow cytometry analysis showing the percentage of α‐SMA‐positive cells following SD‐208 treatment. (F) Extracellular collagen secretion assay measuring total soluble collagen in conditioned media from SD‐208‐treated and control fibroblasts. (G) Representative immunofluorescence images of α‐SMA and fibronectin expression in cardiac fibroblasts treated with SD‐208 or vehicle control. Cells were counterstained with DAPI. Scale bar; 100 µm. (H, I) Quantitative analysis of α‐SMA (H) and fibronectin (I) fluorescence intensity normalized to cell number. All data represent mean ± SEM, *n* = 3 independent biological replicates. *p < 0.05, **p < 0.01, ***p < 0.001, ****p < 0.0001 compared to vehicle control by unpaired *t*‐test.

Having established the phenotypic specification of these cells isolated from HCM patients, we next investigated the anti‐fibrotic effects of SD‐208 treatment. Primary cardiac fibroblasts were treated with SD‐208 (3 µM) for 96 h, and myofibroblast activation markers were assessed using qRT‐PCR analysis (Figure 1A). SD‐208 treatment resulted in significant downregulation of multiple fibroblast activation markers compared to vehicle‐treated control. Expression of *ACTA2* was reduced by >90%, while collagen genes *COL1A1* and *COL3A1* were similarly suppressed. Additionally, SD‐208 treatment significantly reduced expression of the matricellular protein periostin (*POSTN*) and fibroblast activation protein (*FAP*). The pro‐inflammatory cytokines *IL‐6* and *IL‐11*, which are characteristically elevated in activated myofibroblasts, were also markedly reduced following SD‐208 treatment.

To validate these transcriptional changes at the protein level, western blot analysis was performed on whole cell lysates from SD‐208 and vehicle‐treated control cardiac fibroblasts (Figure 1B). Consistent with the gene expression data, SD‐208 treatment led to substantial reduction in α‐SMA protein abundance, with densitometric quantification revealing an approximately 70% decrease compared to controls (Figure 1C). Similarly, COL1A1 protein levels were significantly reduced following SD‐208 treatment (Figure 1D), demonstrating that the transcriptional changes translated into corresponding decreases in protein abundance.

The reduction in myofibroblast markers was further confirmed using flow cytometry analysis to assess the proportion of α‐SMA‐positive cells in the culture (Figure 1E). SD‐208 treatment resulted in a marked decrease in the percentage of α‐SMA‐positive cells, from approximately 65% in control cultures to 25% following treatment, indicating a substantial reduction in the activated myofibroblast population.

To assess whether the observed changes in collagen gene expression translated to functional alterations in extracellular matrix production, we quantified secreted collagen in conditioned media using a colorimetric collagen assay (Figure 1F). SD‐208 treatment led to a significant reduction in extracellular collagen secretion, with treated cells producing approximately 40% less collagen compared to controls. This confirmed that SD‐208 effectively reduced the fibrotic capacity of the cardiac myofibroblasts.

Finally, immunofluorescence microscopy was employed to visualise the spatial distribution and abundance of key myofibroblast markers (Figure 1G). Untreated cardiac fibroblasts exhibited robust α‐SMA expression organised into characteristic stress fibres, indicative of the contractile myofibroblast phenotype. Following SD‐208 treatment, α‐SMA immunoreactivity was markedly reduced, with quantitative analysis showing an approximately 50% decrease in fluorescence intensity (Figure 1H). Similarly, fibronectin expression, another key marker of activated fibroblasts, was significantly diminished following SD‐208 treatment (Figure 1I). The immunofluorescence findings corroborated the western blot and flow cytometry data, providing additional confirmation of SD‐208's anti‐fibrotic effects.

Collectively, these analyses suggest that SD‐208 effectively attenuates the activated myofibroblast phenotype in primary human cardiac fibroblasts derived from HCM patients. The compound reduced expression of fibroblast activation markers at both mRNA and protein levels, decreased the proportion of activated cells in culture, and functionally reduced extracellular matrix production. These findings confirm the expected anti‐fibrotic properties of SD‐208 in the context of human cardiac pathology and establish the foundation for investigating additional cellular effects of this ALK5 inhibitor.

### 3.3 Optimisation and Characterisation of Small Extracellular Vesicle Isolation From Primary Cardiac Fibroblasts

**FIGURE 2:**
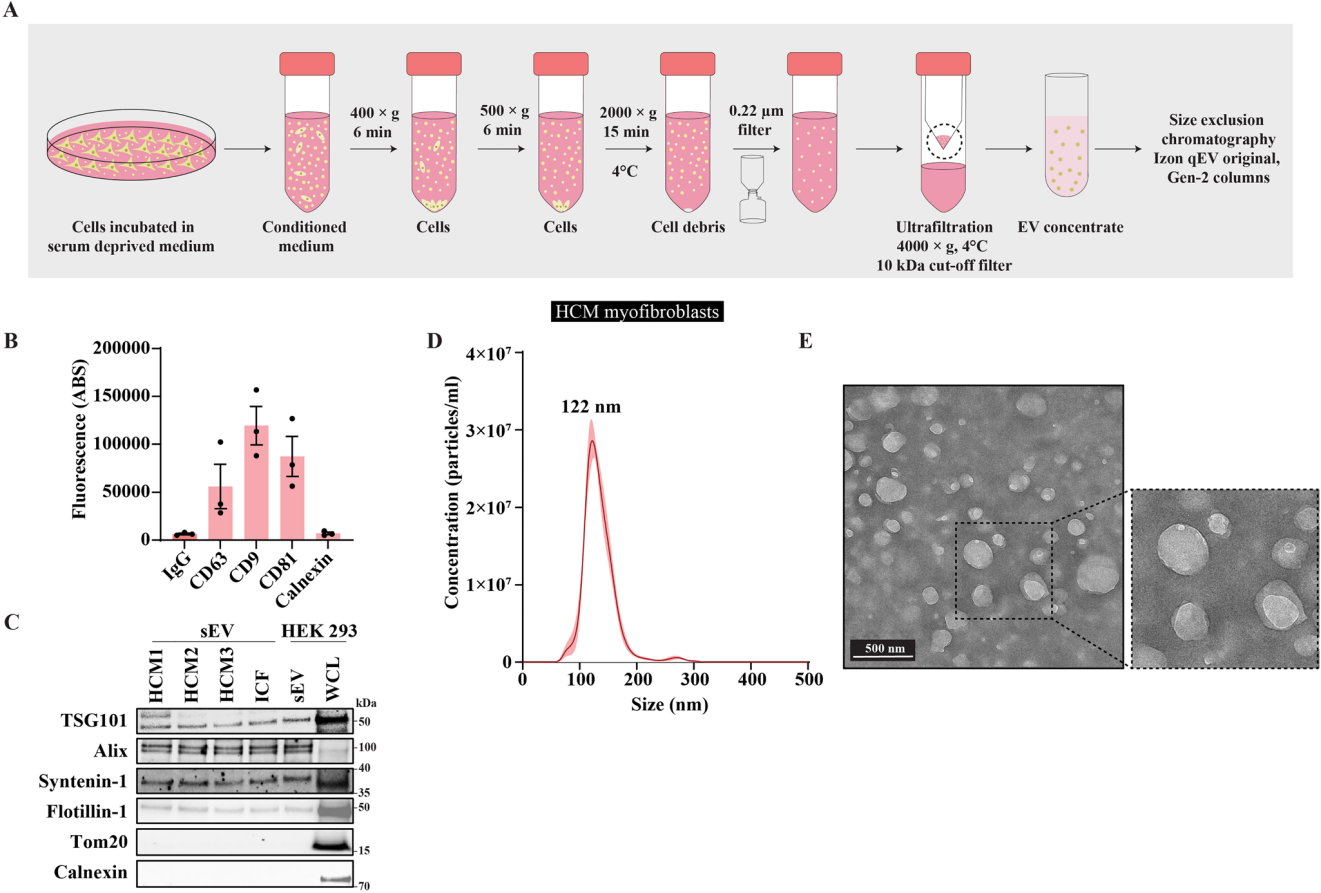
Isolation and characterisation of small extracellular vesicles from primary human cardiac fibroblasts. (A) Schematic of sEV isolation protocol using sequential centrifugation, ultrafiltration, and size exclusion chromatography. (B) Immunophenotyping showing enrichment of EV markers CD9, CD63, CD81, and depletion of calnexin. (C) Western blotting confirmed the presence of luminal sEV markers and depletion of cell abundant proteins in EV preparations. WCL: whole cell lysate. (D) Nanoparticle tracking analysis showing size distribution with modal diameter of 122 nm. (E) Transmission electron microscopy revealing characteristic EV morphology. Scale bar; 100 nm (main).

We next sought to investigate EV secretion from these cells. EV isolation from cardiac fibroblast conditioned medium involved sequential low‐speed centrifugation steps to remove cells and debris, followed by 0.22 µm filtration and ultrafiltration to concentrate the samples (Figure 2A). Size exclusion chromatography enabled further purification, with particle number and protein concentration analysis of all 24 collected fractions to determine optimal sEV recovery. Across three independent cardiac fibroblast isolations from different HCM patients, we consistently observed the highest particle numbers in fractions 7–9, with minimal protein contamination in these fractions (Figure S3A–F). This pattern was reproducible across all patient samples, validating the selection of fractions 7–9 for pooling and downstream analysis.

To verify successful EV isolation and assess preparation quality, we performed characterisation using complementary approaches in accordance with MISEV 2023 guidelines (Welsh et al. 2024). Immunophenotyping analysis revealed robust enrichment of classical sEV surface markers CD9, CD81, and CD63, with CD9 showing the highest signal intensity (Figure 2B). Importantly, the endoplasmic reticulum marker calnexin was barely detectable in the EV preparations, confirming minimal contamination with intracellular organelles. Western blotting further confirmed presence of luminal sEV markers ALIX, TSG101, Syntenin‐1, Flotillin‐1 in EVs from HCM patient derived primary cells, ICF and HEK293 cells. Cell abundant proteins Tom20 and calnexin while detected in HEK293 whole cell lysate, could not be detected in the EVs, reflecting the purity of sEV preparations (Figure 2C).

NTA demonstrated that the isolated particles exhibited size characteristics consistent with sEVs, with a modal diameter of 122 nm and the majority of particles falling within the 50–200 nm range (Figure 2D). TEM analysis further confirmed the vesicular nature of the isolated particles, revealing vesicular morphology consistent with extracellular vesicles following negative staining (Figure 2E). The particles displayed intact structures with polydisperse particle size distribution, providing morphological validation of successful sEV isolation.

Together, these characterisation studies established a reliable and reproducible protocol for isolating pure sEVs from primary human cardiac fibroblasts. The combination of biochemical, physical, and morphological analyses confirmed that our preparations met current MISEV guidelines (Welsh et al. 2024) for EV characterisation, providing a robust experimental foundation for investigating the modulatory effects of pharmacological compounds on EV secretion.

### 3.4 SD‐208 Markedly Reduces sEV Secretion From HCM Cardiac Fibroblasts Without Compromising Cellular Health

We next investigated whether SD‐208 might modulate EV secretion. To examine this possibility, primary cardiac fibroblasts were treated with SD‐208 (3 µM) for 96 h under serum‐starved conditions, and sEVs were isolated from conditioned media for quantitative comparison with vehicle‐treated controls.

**FIGURE 3:**
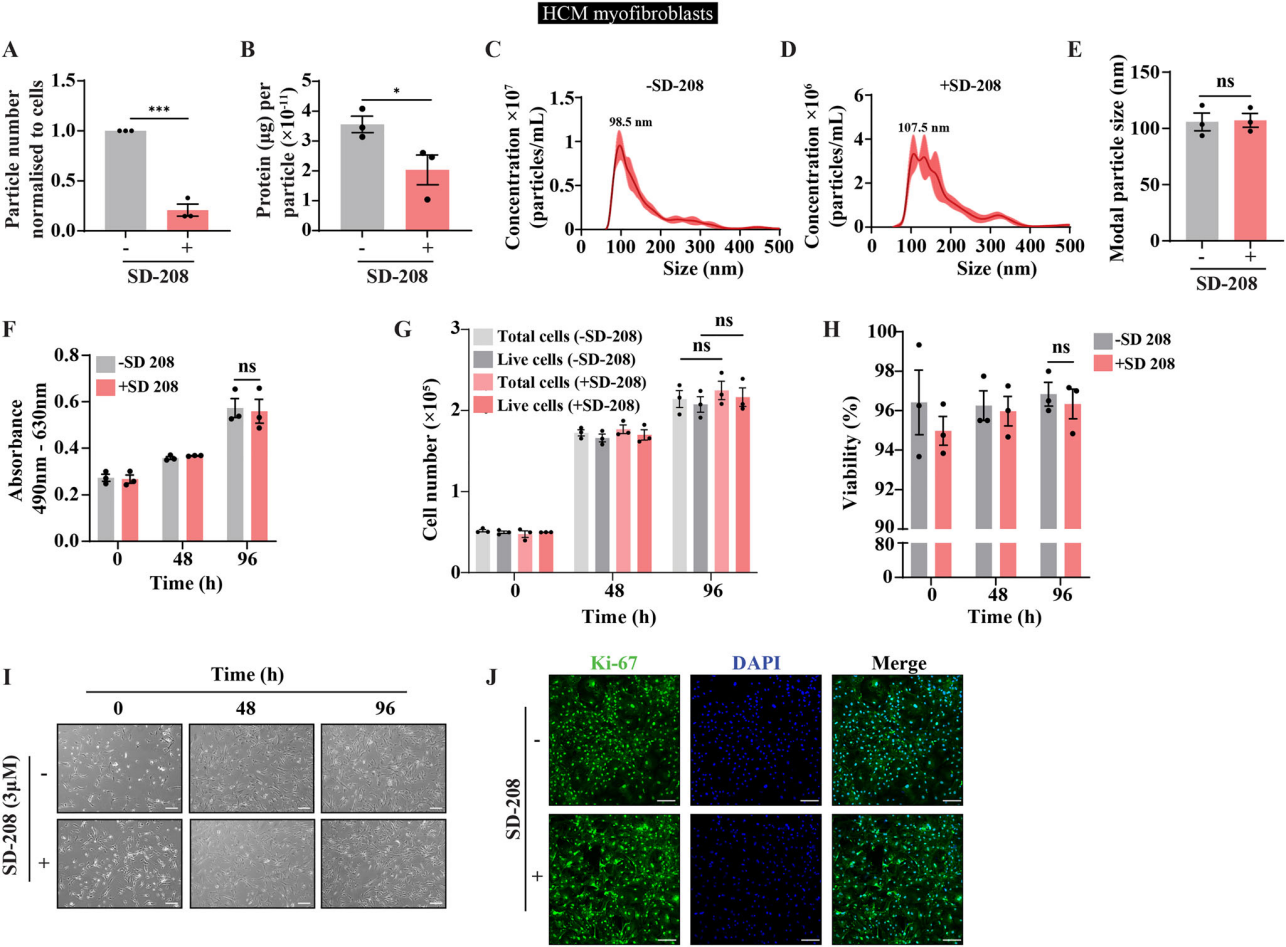
SD‐208 reduces EV secretion from primary cardiac fibroblasts without affecting cellular viability. (A) NTA showing reduced particle concentration per cell following SD‐208 treatment (3 µM, 96 h). (B) Protein content per particle in isolated EVs. (C, D) NTA generated histograms for particle size distribution and concentration for sEVs secreted by primary cardiac fibroblasts without (C) and with (D) SD‐208 treatment. (E) Modal EV size remains unchanged between control and SD‐208‐treated groups. (F‐H) Cell viability assessments: (F) metabolic activity (MTS assay), (G, H) viability (trypan blue exclusion), and proliferation (cell number). (I) Phase contrast microscopy showing cell morphology and density at 0, 48, and 96 h. Scale bar; 100 µm. (J) Confocal immunofluorescence of Ki‐67 proliferation marker. Scale bar; 200 µm. Data represent mean ± SEM, *n* = 3 independent biological replicates. *p < 0.05, **p < 0.01 by unpaired *t*‐test.

NTA revealed a profound impact of SD‐208 treatment on sEV secretion. The number of particles secreted per cell was significantly reduced (approximately 75% reduction) following SD‐208 treatment compared to vehicle controls (Figure 3A). Moreover, analysis of sEV protein content demonstrated that SD‐208‐treated cells also released vesicles with reduced protein per particle (Figure 3B), suggesting that the compound affects both the quantity of sEVs secreted and their protein cargo loading. Importantly, despite these marked quantitative changes, the modal size of secreted EVs remained unchanged between control and SD‐208‐treated groups. Size distribution histograms further confirmed that both populations of sEVs exhibited similar size profiles within the expected sEV range (Figure 3C–E), indicating that SD‐208 does not alter the fundamental size characteristics of secreted vesicles but rather modulates their abundance and cargo content.

Given the substantial reduction in sEV secretion observed, we next sought to determine whether this effect was due to compromised cellular health or represented a specific modulatory action on sEV secretion pathways (Catalano and O'Driscoll 2020; Essandoh et al. 2015; Li et al. 2022). We systematically assessed multiple parameters of cell viability, metabolic activity, and proliferative capacity. MTS assay analysis revealed no significant differences in metabolic activity between SD‐208‐treated and control cells (Figure 3F), demonstrating that mitochondrial function and cellular energy metabolism remained intact. Trypan blue exclusion assays confirmed that cell viability was unaffected by SD‐208 treatment, with both groups maintaining comparable viability throughout the experimental period. Additionally, cell proliferation assessed by total cell number showed no impairment in SD‐208‐treated cultures (Figure 3G,H), ruling out anti‐proliferative effects as a cause of reduced EV secretion.

Morphological assessment provided further evidence of preserved cellular health. Phase contrast microscopy examination at 0, 48, and 96 h timepoints revealed no observable differences in cell morphology, density, or confluence between SD‐208‐treated and control cardiac fibroblasts (Figure 3I). The cells maintained their characteristic elongated morphology and achieved similar confluence regardless of treatment status. To more sensitively assess proliferative activity, we performed immunofluorescence staining for the proliferation marker Ki‐67. Confocal microscopy revealed similar Ki‐67 expression patterns and nuclear distribution between control and SD‐208‐treated cells (Figure 3J), confirming that proliferative capacity was preserved at the single‐cell level.

To further assess the generalisability of this effect, we extended our analysis to an independent primary human cardiac fibroblast population (PromoCell; dilated cardiomyopathy donor). Consistent with our findings in HCM‐derived fibroblasts, SD‐208 treatment significantly reduced sEV secretion without affecting cell viability, proliferation, or metabolic activity (Figure S4), confirming that this effect is conserved across independent human cardiac fibroblast populations with distinct pathological backgrounds.

These comprehensive analyses support a model in which SD‐208 specifically suppresses sEV secretion from primary cardiac fibroblasts without compromising cellular viability, metabolic function, or proliferative capacity. The preservation of multiple cellular health parameters demonstrates that the observed reduction in EV release represents a targeted effect on vesicle secretion mechanisms rather than a secondary consequence of any detectable impairment of the cellular dysfunction or stress parameters assessed.

### 3.5 SD‐208 Reduces sEV Secretion in Cardiac Fibroblasts Independently of Anti‐Fibrotic Effects

To determine whether the observed reduction in sEV secretion was dependent on the anti‐fibrotic effects of SD‐208 or represented an independent mechanism, we investigated commercially available immortalised cardiac fibroblasts (ICFs). These cells provide a complementary model system to primary HCM cardiac fibroblasts, allowing us to assess whether SD‐208's effects on EV secretion require an activated myofibroblast phenotype.

We first characterised the baseline activation status of ICFs and their response to TGF‐β stimulation and SD‐208 treatment. qRT‐PCR analysis revealed that unstimulated ICFs exhibited relatively low expression of myofibroblast activation markers compared to primary HCM fibroblasts (Figure S5A). Treatment with recombinant human TGF‐β1 (5 ng/mL and 10 ng/mL) induced upregulation of multiple fibrotic markers including *ACTA2*, *COL1A1*, *FAP*, *POSTN*, *IL‐6*, and *IL‐11*, confirming that these cells retain the capacity to undergo myofibroblast activation (Figure S5B). SD‐208 treatment (3 µM) modestly reduced the mRNA levels of these activation markers in the non‐activated baseline state, though the effect was less pronounced than observed in the already‐activated primary HCM fibroblasts (Figure 1A).

Flow cytometry analysis provided additional characterisation of the cellular phenotype under different treatment conditions (Figures S5C and S6). Consistent with the mRNA level data, unstimulated ICFs showed relatively low baseline expression of α‐SMA, fibronectin, and collagen‐1. TGF‐β stimulation significantly increased the percentage of cells positive for these myofibroblast markers, with both 5 and 10 ng/mL doses producing robust activation (Figure S5C). SD‐208 treatment of non‐activated ICFs resulted in modest reductions in marker expression, confirming that these cells maintained a relatively quiescent phenotype under baseline culture conditions.

**FIGURE 4:**
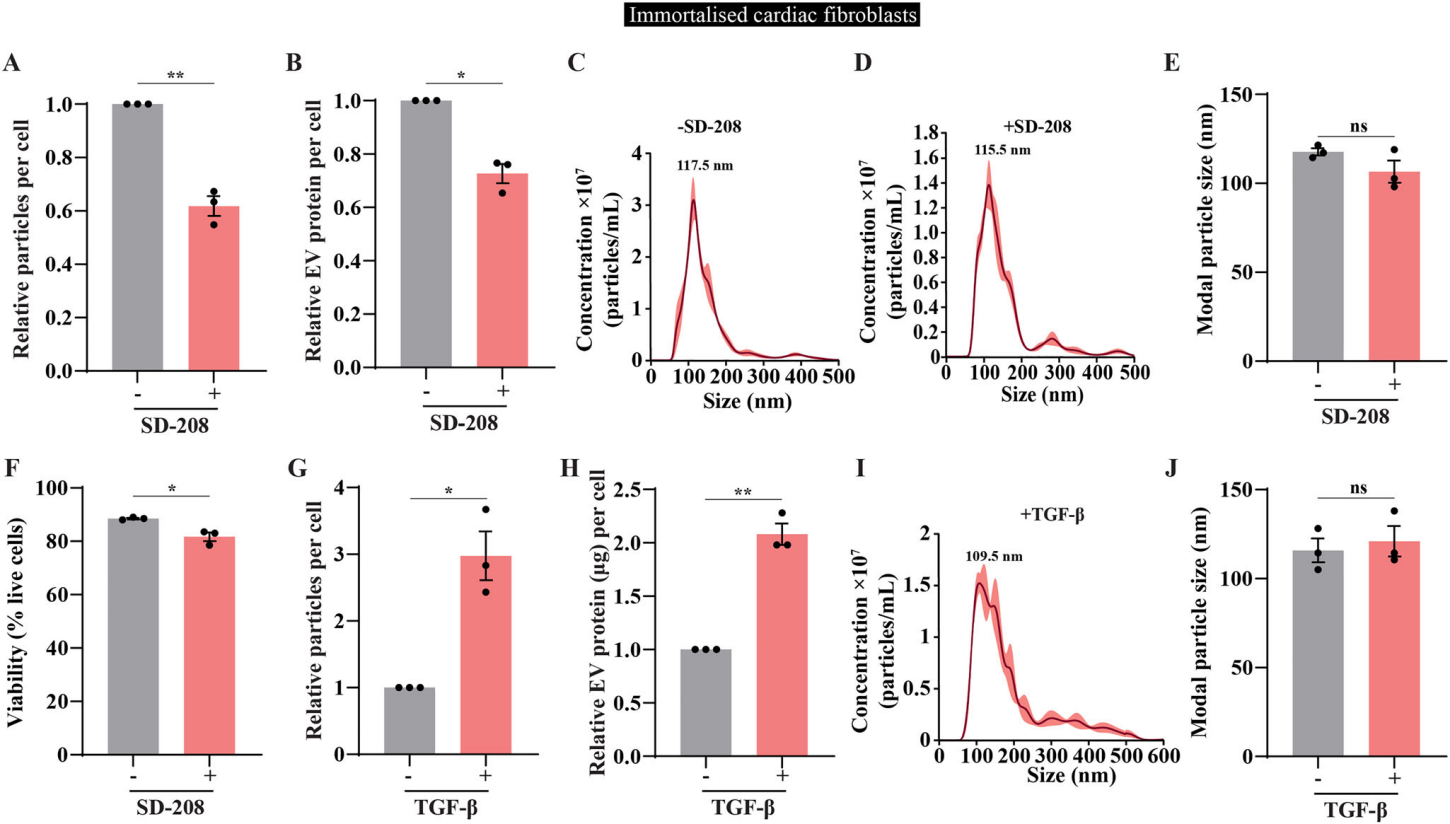
SD‐208 reduces sEV secretion from immortalised cardiac fibroblasts. (A, B) Quantification of sEV secretion from ICFs treated with SD‐208 (3 µM, 96 h) compared to vehicle control. (A) Particle concentration per cell measured by nanoparticle tracking analysis (NTA) showing significant reduction with SD‐208 treatment. (B) EV protein content per cell demonstrating decreased protein secretion in SD‐208‐treated cultures. (C) Size distribution analysis of secreted sEVs. Representative NTA histograms showing particle size distribution for control (C) and SD‐208‐treated (D) cells. (E) Modal particle size showing no significant difference between groups. (F) Cell viability assessed by trypan blue exclusion. (G, J) Effects of TGF‐β stimulation on sEV secretion. (G) particle concentration per cell showing increased sEV secretion and (H) EV protein per cell following TGF‐β1 treatment (10 ng/mL, 96 h). (I) Representative NTA histogram for TGF‐β‐stimulated cells. (J) Modal particle size remains unchanged with TGF‐β treatment. Data represent mean ± SEM, *n* = 3 independent biological replicates. *p < 0.05, **p < 0.01 unpaired *t*‐test. ns, not significant.

Having established the activation characteristics of ICFs, we next examined the effects of SD‐208 and TGF‐β on sEV secretion. Critically, despite the modest effects of SD‐208 on fibroblast activation markers in these non‐activated cells, the compound produced a marked reduction in sEV secretion. NTA revealed that SD‐208 treatment significantly reduced both particle concentration per cell (Figure 4A) and EV protein content per cell (Figure 4B) compared to controls. This reduction in sEV secretion occurred despite minimal changes in the activation status of these already non‐activated fibroblasts, demonstrating that SD‐208's effect on EV release is independent of its anti‐fibrotic properties.

Analysis of EV size distribution confirmed that SD‐208 did not alter the fundamental biophysical characteristics of secreted vesicles. Size distribution histograms showed similar profiles between control and SD‐208‐treated groups (Figure 4C,D), with modal particle sizes of 117.5 and 115.5 nm, respectively (Figure 4E), falling within the expected range for small EVs. This consistency in size distribution indicates that SD‐208 modulates vesicle quantity rather than affecting the vesicle formation machinery that determines particle size.

In contrast to SD‐208's inhibitory effect, TGF‐β stimulation enhanced sEV secretion in parallel with inducing myofibroblast activation. Treatment with TGF‐β1 (10 ng/mL) increased both particle concentration per cell (Figure 4G) and sEV protein per cell (Figure 4H) compared to unstimulated controls. NTA size distribution analysis showed that TGF‐β‐stimulated cells secreted vesicles with a modal size of 109 nm (Figure 4I,J), similar to controls, indicating that increased activation enhances EV secretion without altering vesicle size characteristics. This finding is consistent with previous reports linking fibroblast activation to enhanced EV release and establishes that activated myofibroblasts are more prolific EV producers than their quiescent counterparts. Assessment of cellular health parameters confirmed that the observed changes in sEV secretion were not consequences of compromised cell viability (Figure 4F) as SD‐208 treatment had no significant effect on cell viability as assessed by trypan blue exclusion, demonstrating that altered EV secretion represents specific biological responses rather than secondary effects of cellular dysfunction.

These findings in ICF provide critical insight into the mechanism of SD‐208's action on EV secretion. The observation that SD‐208 substantially reduces sEV release even when it produces minimal changes in fibroblast activation markers demonstrates that its effect on vesicle secretion is mechanistically independent of ALK5‐mediated anti‐fibrotic signalling. Conversely, TGF‐β stimulation increases both myofibroblast activation and sEV production, suggesting that while fibrotic activation can enhance sEV secretion, SD‐208's inhibitory effect on sEV release does not require suppression of this activation state. This dissociation between anti‐fibrotic activity and EV inhibition suggests that SD‐208 may have dual, independent actions on cardiac fibroblast biology.

### 3.6 SD‐208 Reduces sEV Secretion in Non‐Fibrotic HEK293 Cells Independent of Fibrotic Signalling

To definitively establish that SD‐208's inhibitory effect on sEV secretion occurs independently of cardiac fibrotic signalling pathways, we investigated the compound's effects in HEK293 cells, a widely used non‐fibrotic embryonic kidney cell line that lacks the TGF‐β‐responsive fibrotic machinery characteristic of cardiac fibroblasts. HEK293 cells are commonly employed in EV research due to their robust vesicle secretion and well‐characterised biology, making them an ideal model to isolate SD‐208's effects on vesicle trafficking from any confounding anti‐fibrotic actions (Stepanenko and Dmitrenko 2015; Liu et al. 2016).

**FIGURE 5:**
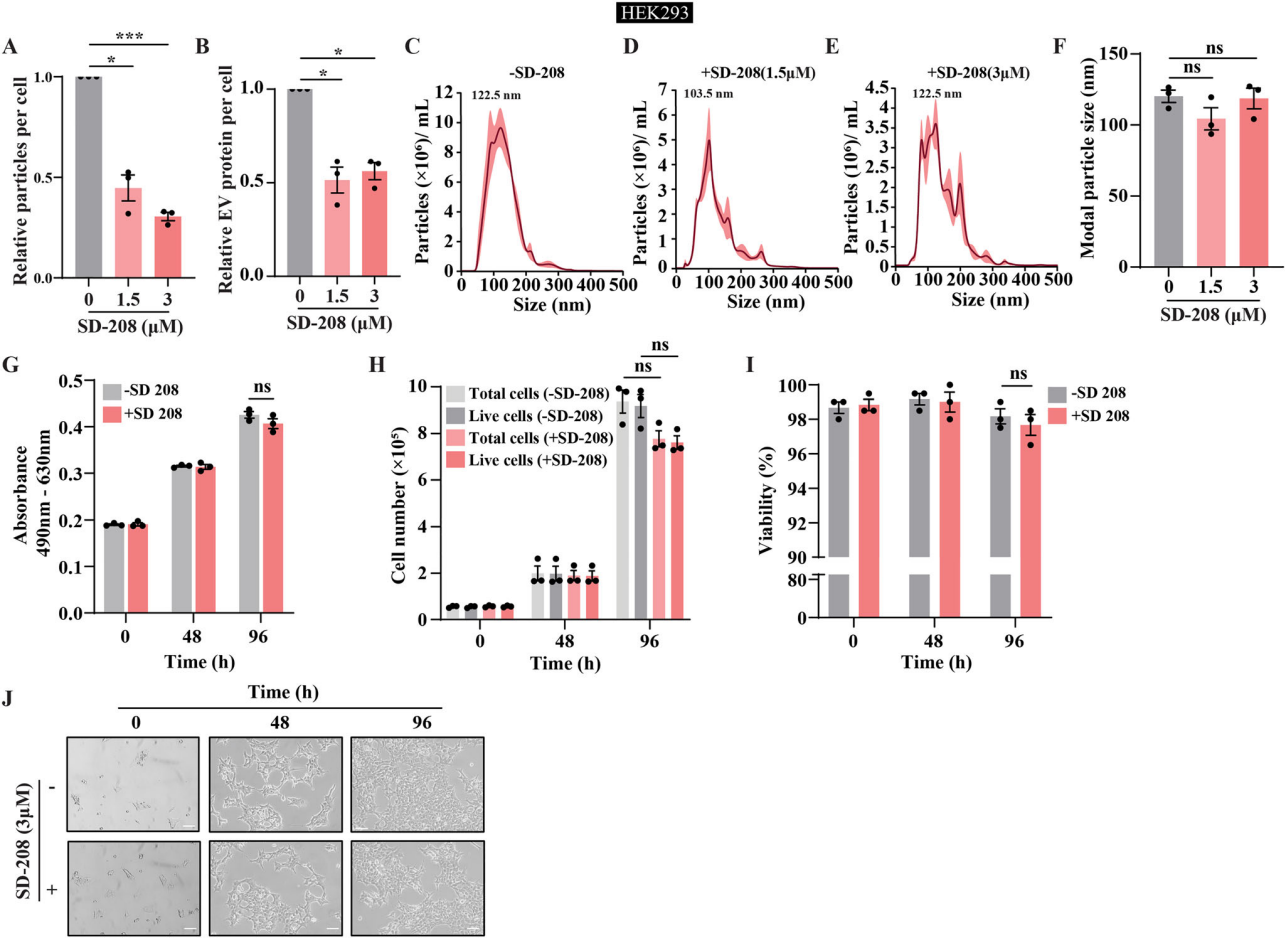
SD‐208 reduces sEV secretion from HEK293 cells without affecting cellular viability or proliferation. (A, B) Dose‐dependent effects of SD‐208 on sEV secretion from HEK293 cells treated for 96 h. (A) Particle concentration per cell showing significant reduction with 1.5 µM and 3 µM SD‐208. (B) EV protein content per cell demonstrating dose‐dependent decrease. (C–F) Size distribution analysis of secreted sEVs. Representative NTA histograms for vehicle control (C), 1.5 µM SD‐208 (D), and 3 µM SD‐208 (E). (F) Modal particle size showing no significant changes across treatment groups. (G) MTS assay measuring cellular metabolic activity at 0, 48, and 96 h showing no significant differences between control and SD‐208‐treated cells. (H) Total cell number and live cell counts over time demonstrating preserved proliferative capacity with SD‐208 (3 µM) treatment. (I) Cell viability assessed by trypan blue exclusion showing consistently high viability (>97%) at all timepoints in both control and SD‐208‐treated cultures. (J) Representative phase contrast microscopy images showing cell morphology and density at 0, 48, and 96 h for control and SD‐208‐treated (3 µM) HEK293 cells. 0 h (J‐0) represents the baseline timepoint measured 24 h after cell seeding and immediately following SD‐208 addition. Scale bar; 100 µm. Data represent mean ± SEM, *n* = 3 independent biological replicates. *p < 0.05, ***p < 0.001 by, one‐way ANOVA with Tukey's post‐hoc test (A, B, F) or two‐way ANOVA (G‐I). ns, not significant.

HEK293 cells were treated with SD‐208 at two concentrations (1.5 and 3 µM) for 96 h under serum‐starved conditions to match the treatment paradigm used in fibroblast studies. NTA revealed a dose‐dependent reduction in sEV secretion following SD‐208 treatment. Particle concentration per cell was significantly reduced with both 1.5 and 3 µM SD‐208 compared to vehicle‐treated controls (Figures 5A and S7A–D), with the 3 µM dose producing approximately 70% reduction in particle secretion. Similarly, EV protein content per cell was significantly decreased upon treatment with SD‐208 at both the doses (Figure 5B), confirming that SD‐208 inhibits sEV release in this non‐fibrotic cell type.

Analysis of EV size distribution demonstrated that SD‐208 treatment did not alter the fundamental characteristics of secreted vesicles. Representative size distribution histograms from control cells (Figure 5C), 1.5 µM SD‐208‐treated cells (Figure 5D), and 3 µM SD‐208‐treated cells (Figure 5E) showed similar profiles, with modal particle sizes of 122.5, 103.5, and 122.5 nm, respectively (Figure 5F). Although modest variations in modal size were observed, these differences were not statistically significant and all populations remained within the characteristic size range for small EVs (50–200 nm), indicating that SD‐208 affects vesicle quantity rather than the biogenesis machinery determining vesicle size.

To ensure that the observed reduction in sEV secretion was not a consequence of compromised cellular health or metabolic dysfunction, we performed viability and proliferation assessments across the 96 h treatment period. MTS assay analysis revealed that SD‐208 treatment did not significantly affect cellular metabolic activity at any timepoint examined (0, 48, and 96 h) (Figure 5G). Both control and SD‐208‐treated cells showed comparable metabolic activity, with the expected increase over time as cells proliferated, demonstrating that mitochondrial function and cellular energy metabolism remained intact.

Cell counting analysis confirmed that SD‐208 treatment did not impair cell proliferation. Both total cell number and live cell number increased comparably between control and SD‐208‐treated groups over the 96 h period (Figure 5H). At 48 and 96 h timepoints, there were no significant differences in cell number between treatment groups, indicating normal proliferative capacity. Trypan blue exclusion assays demonstrated that cell viability remained consistently high (>97%) in both control and SD‐208‐treated cultures at all timepoints (Figure 5I), confirming that the compound did not induce cytotoxicity or compromise cell membrane integrity.

Morphological assessment provided additional evidence of preserved cellular health. Phase contrast microscopy at 0, 48, and 96 h revealed no observable differences in cell morphology, density, or growth patterns between SD‐208‐treated and control HEK293 cells (Figure 5J). Both groups displayed typical HEK293 morphology with characteristic epithelial‐like appearance and reached similar confluence by 96 h, further confirming that cellular function was maintained throughout the treatment period.

The findings in HEK293 cells support the notion that SD‐208's inhibitory effect on sEV secretion can occur independently of canonical anti‐fibrotic pathways. Unlike cardiac fibroblasts, HEK293 cells lack the TGF‐β‐responsive transcriptional machinery that mediates fibrotic activation and do not express key components of the cardiac fibrotic program. The observation that SD‐208 produces comparable reductions in sEV secretion in both cell types—despite their vastly different biological contexts—demonstrates that the compound acts through a conserved mechanism related to vesicle trafficking rather than through cell‐type‐specific fibrotic signalling pathways.

Moreover, the sEV secretion inhibition in HEK293 cells, mirroring observations in cardiac fibroblasts, suggests a direct pharmacological interaction with cellular machinery governing vesicle secretion. The preservation of cell viability, metabolic activity, and proliferative capacity across all treatment conditions rules out the possibility that reduced EV secretion results from general cellular stress or dysfunction. Instead, these data support a model in which SD‐208 specifically targets vesicle trafficking and secretion machinery that is conserved across diverse cell types.

Together with the findings in non‐activated cardiac fibroblasts (Section 3.5), these results in HEK293 cells demonstrate that SD‐208 functions as a pharmacological inhibitor of sEV secretion through a mechanism independent of ALK5‐mediated anti‐fibrotic signalling. This dual functionality—anti‐fibrotic activity in fibroblasts coupled with vesicle secretion inhibition across multiple cell types—positions SD‐208 as a unique small molecule tool for both mechanistic investigation of EV biology and potential therapeutic applications in diseases where EV‐mediated intercellular communication drives pathology.

### 3.7 Proteomic Profiling Reveals Dysregulation of Vesicle Trafficking and Lysosomal Pathways

**FIGURE 6:**
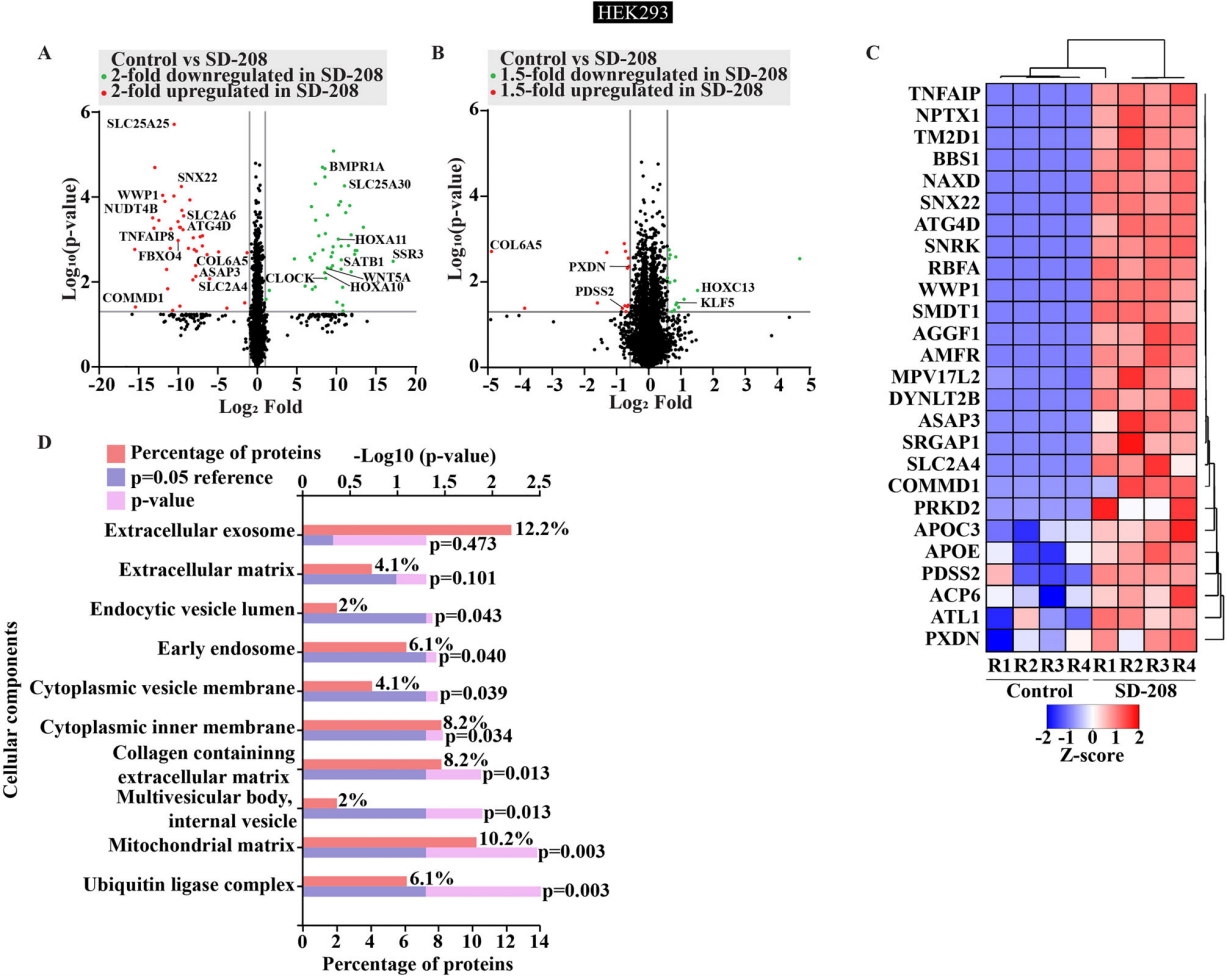
Differential protein expression and cellular component enrichment in SD‐208‐treated HEK293 cells. (A) Volcano plot showing differential protein expression between control and SD‐208‐treated cells. Proteins with ≥2‐fold downregulation (right) and upregulation (left) are highlighted in red and green, respectively. Selected protein names are labelled. (B) Volcano plot highlighting proteins with ≥1.5‐fold changes. Horizontal dashed line represents *p* = 0.05. (C) Heatmap showing hierarchical clustering of proteins involved in vesicle trafficking, endosomal sorting, EV secretion, lysosomal degradation, autophagy, and mitochondrial metabolism across biological replicates (R1–R4). Colour scale represents Z‐score normalized protein abundance. (D) Gene ontology analysis of cellular component enrichment showing percentage of proteins (red bars) and −log_10_ *p* value (purple bars). Data represent mean, *n* = 4 independent biological replicates.

To gain comprehensive molecular insights into the mechanisms underlying SD‐208's inhibitory effects on sEV secretion, we performed quantitative proteomic analysis of HEK293 cells treated with SD‐208 (3 µM, 96 h) compared to vehicle controls (Figure S8A). This unbiased approach aimed to identify global changes in protein abundance that might explain how SD‐208 disrupts vesicle secretion while preserving cellular viability and metabolic function (Figure 6A, Table S5, S10–S13). Principal component analysis (PCA) demonstrated clear separation between control and SD‐208‐treated samples (Figure S8B), indicating that SD‐208 treatment induced substantial and reproducible changes in the cellular proteome.

Across the dataset, 7231 proteins were commonly detected in both conditions, with 115 proteins identified only in controls and 105 proteins uniquely detected following SD‐208 treatment (Figure S8C). Differential expression analysis revealed substantial proteomic remodelling. Using a stringent ≥2‐fold threshold, 15 proteins were significantly upregulated and 10 were downregulated in SD‐208‐treated cells (Figure S8D). A 1.5‐fold threshold identified a broader set of changes, including 98 upregulated and 81 downregulated proteins (Figure S8E). Volcano plot analysis (Figure 6A,B) highlighted upregulation of collagens (COL6A5, COL6A3), solute carriers (SLC25A25, SLC2A6, SLC2A1), and proteins involved in lipid metabolism and vesicle trafficking (WWP1, NUDT4B, TNFAIP8, FBXO4, COMMD1). Notably, upregulated proteins included components of vesicle secretion machinery, consistent with the observed reduction in sEV release and their intracellular retention (Burkhead et al. 2009; Niture et al. 2020; Spagnol et al. 2016; Nozaki et al. 2025; Choi et al. 2023; Cullen 2008; Caviston et al. 2007; Datta et al. 2017; Dutta and Donaldson 2015; Yorimitsu et al. 2014; Yamazaki et al. 2013). Conversely, proteins downregulated in SD‐208‐treated cells included BMPR1A, SLC25A30, and various metabolic and signalling proteins, suggesting reduced activity of certain cellular pathways. However, the 1.5‐fold threshold analysis also revealed additional dysregulated proteins including PXDN (peroxidasin), PDSS2, HOXC13, and KLF5 (Peng et al. 2008; Quinzii et al. 2013; Péterfi et al. 2009; Li et al. 2023; Nagai et al. 2005).

Hierarchical clustering revealed coordinated changes across multiple interconnected pathways (Figure 6C). Proteins involved in endosomal trafficking, EV biogenesis, lysosomal degradation, autophagy, and mitochondrial metabolism formed distinct expression clusters, suggesting that SD‐208 perturbs the broader vesicle trafficking network rather than inhibiting a single pathway.

Gene ontology analysis provided strong mechanistic insights. Cellular component enrichment demonstrated significant overrepresentation of proteins associated with MVB internal vesicles (*p* = 0.013) and cytoplasmic vesicle membranes, directly supporting the intracellular accumulation of MVBs observed experimentally. Mitochondrial matrix components were also enriched (*p* = 0.003), consistent with increased TOM20 expression and altered mitochondrial architecture (Farmer et al. 2017). Ubiquitin ligase complex components showed robust enrichment (*p* = 0.003), suggesting increased ubiquitination and routing of proteins through degradative pathways (Figure 6D) (Shelke et al. 2023). Additional enriched components included cytoplasmic inner membrane (8.2%, *p* = 0.034), cytoplasmic vesicle membrane (4.1%, *p* = 0.039), and collagen‐containing extracellular matrix (8.2%, *p* = 0.013), reflecting widespread reorganisation of vesicular compartments and altered ECM protein handling despite overall reduction in collagen secretion.

Extended analysis (Figure S9A) further revealed enrichment of ER and ER‐to‐Golgi transport machinery, early endosomes, and proteins normally associated with extracellular exosomes—consistent with the intracellular retention of cargo that would otherwise be secreted. Comparative component analysis (Figure S9B) showed depletion of proteins associated with extracellular vesicles and endocytic vesicle membranes, but strong enrichment of late endosomes, recycling endosomes, MVBs, autophagosome membranes, clathrin‐coated vesicles, and ubiquitin machinery. These findings collectively indicate that SD‐208 drives vesicle accumulation within late endosomal and degradative compartments.

Biological process enrichment supported these conclusions (Figure S10A,B). As expected, TGF‐β receptor signalling was significantly depleted, consistent with ALK5 inhibition. Processes related to vesicle trafficking—including plasma membrane–to–endosome transport and endosome–to–lysosome transport—were strongly enriched, indicating a shift toward lysosomal routing. Ubiquitin‐dependent protein catabolic processes were among the most highly enriched, reinforcing increased degradative trafficking. Autophagy‐related pathways were also broadly enriched, although depletion of autophagosome regulatory processes suggests a complex modulation of autophagic flux rather than uniform activation (Song et al. 2019; Underwood et al. 2020). Vesicle trafficking processes, particularly endosome‐to‐lysosome transport, were strongly enriched following SD‐208 treatment, which may be consistent with reduced EV secretion. Ubiquitin‐dependent protein catabolism (∼300‐fold) and intrinsic apoptotic signalling/positive regulation of protein ubiquitination (214‐fold) indicate enhanced routing of cargo to degradative pathways and activation of protein quality‐control mechanisms, without affecting cell viability. ALK5 inhibition depleted MAPK, JNK, SMAD, and cell‐cycle pathways, while positive regulation of Wnt signalling and fibroblast proliferation (107.6‐fold) likely represents compensatory adaptation (Clevers and Nusse 2012; Akhmetshina et al. 2012; Carthy et al. 2011). Enrichment of calcium‐ion transport (214‐fold) suggests altered calcium homeostasis, relevant for vesicle fusion.

To determine whether similar proteomic changes occur in the disease‐relevant primary fibroblast model, we performed comparative proteomic profiling of SD‐208‐treated and control primary HCM‐derived myofibroblasts from the same three patient‐derived cultures used in the initial experiments (HCM1–3). This analysis revealed SD‐208‐associated remodelling of proteins linked to vesicle trafficking, endosomal regulation, ubiquitin/proteostasis, autophagy, mitochondrial compartments, and fibrosis‐associated pathways (Figure S11, Tables S9 and S14–S17). Altered trafficking‐associated proteins included VPS37C, STXBP3, TSPAN4, RAB29, RAB3A, RBSN, SNX29, and SORT1, while fibrosis‐ and extracellular matrix‐associated proteins including COL1A1, COL1A2, COL5A1, FN1, TGFB1, SERPINE1, LOX, SPARC, FAP, and CCN2 were reduced or differentially regulated. These findings support the relevance of the SD‐208‐induced trafficking and proteostatic signature in primary HCM‐derived myofibroblasts and provide additional disease‐relevant support for the proposed rerouting of vesicular cargo toward degradative pathways.

Collectively, this comprehensive proteomic analysis supports a model in which SD‐208 may disrupt sEV secretion by modulating vesicle trafficking machinery and promoting lysosomal routing of MVBs without detectable impairment of cell function. The coordinated enrichment of proteins associated with MVBs, late endosomes, lysosomes, autophagosomes, and ubiquitin ligase complexes supports a model in which SD‐208 causes intracellular accumulation of vesicles that would normally be secreted, routing them instead through degradative pathways. Enrichment of mitochondrial and ER‐Golgi trafficking components highlights broader effects on cellular membrane dynamics and organelle communication, consistent with a global rerouting of vesicular cargo within the endo‐lysosomal system.

### 3.8 SD‐208 Disrupts Vesicle Trafficking by Promoting Intracellular CD63 Accumulation and Lysosomal Targeting

Having established that SD‐208 reduces sEV secretion across multiple cell types without compromising cellular viability and having identified proteomic signatures suggesting disrupted vesicle trafficking and enhanced lysosomal targeting, we next sought to directly visualise and quantify these mechanistic changes at the protein and subcellular levels. We focused on CD63, a tetraspanin protein highly enriched in MVBs and commonly used as a marker for intraluminal vesicles destined for secretion as exosomes, alongside markers of lysosomes (LAMP1), autophagosomes (LC3A/B), and mitochondria (TOM20).

**FIGURE 7:**
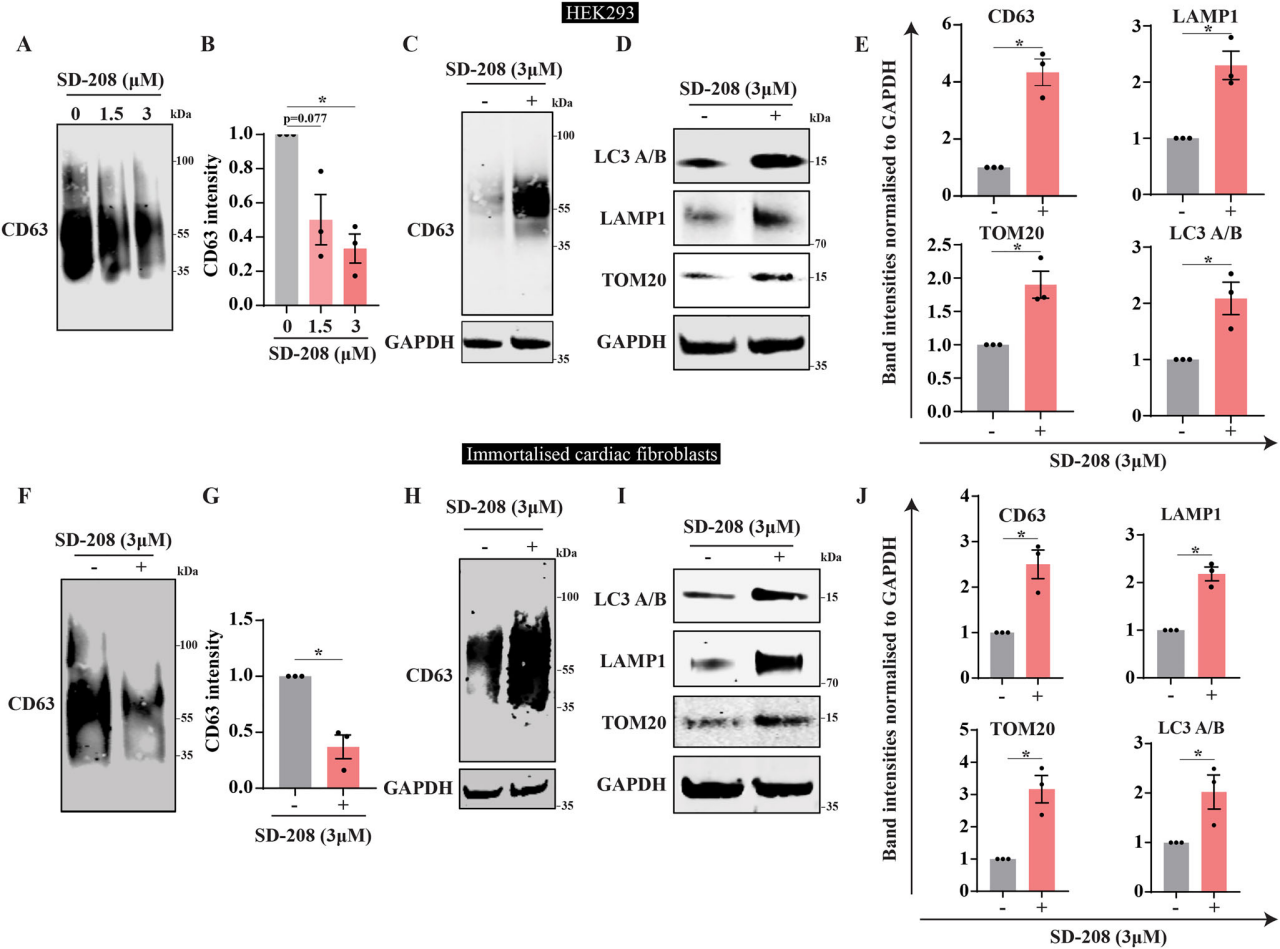
SD‐208 induces intracellular accumulation of CD63 and upregulates lysosomal, autophagic, and mitochondrial markers. (A–E) Western blot analysis in HEK293 cells treated with SD‐208 (1.5 µM or 3 µM, 96 h). (A) Representative western blot for sEVs secreted by equal cell number showing dose‐dependent decrease in sEV CD63 levels following SD‐208 treatment. (B) Densitometric quantification of sEV‐associated CD63. (C) Representative western blot showing increased intracellular CD63 retention or accumulation in SD‐208‐treated cells. (D) Western blot analysis of LC3A/B, LAMP1, TOM20, and GAPDH in whole cell lysates. (E) Quantification of CD63, LAMP1, TOM20, and LC3A/B protein levels normalized to GAPDH. (F–J) Western blot analysis in immortalised cardiac fibroblasts treated with SD‐208 (3 µM, 96 h). (F) Representative western blot for sEVs secreted by equal cell number showing decrease in sEV‐associated CD63 levels following SD‐208 treatment. (G) Densitometric quantification of sEV‐associated CD63. (H) Representative western blot showing increased intracellular CD63 retention or accumulation in SD‐208‐treated cells. (I) Western blot analysis of LC3A/B, LAMP1, TOM20, and GAPDH with and without SD‐208 treatment. (J) Quantification of CD63, LAMP1, TOM20, and LC3A/B protein levels normalized to GAPDH as the housekeeping gene. Data represent mean ± SEM, *n* = 3 independent biological replicates. *p < 0.05, by unpaired t‐test or one‐way ANOVA with Tukey's post‐hoc test.

Western blot analysis and densitometric quantification of sEVs isolated from equal numbers of HEK293 cells treated with SD‐208 (0, 1.5 or 3 µM, 96 h) revealed a dose‐dependent reduction in CD63 levels, consistent with reduced sEV secretion (Figure 7A,B). This was mirrored by intracellular CD63 accumulation upon SD‐208 treatment confirming that vesicles were being retained within cells rather than secreted as confirmed by western blot analysis and densitometric quantification of whole cell lysates from HEK293 cells treated with SD‐208 (3 µM, 96 h) (Figure 7C,E). Analysis of additional trafficking and degradation pathway markers revealed that SD‐208 treatment significantly increased protein levels (approximately 2‐fold increase) of LAMP1, LC3A/B, and TOM20 (Figure 7D,E). This indicates coordinated upregulation of lysosomal, autophagic, and mitochondrial compartments in response to disrupted vesicle trafficking.

To validate these findings in a cardiac‐relevant model, we performed parallel western blot analyses in ICF treated with SD‐208 (3 µM, 96 h). As in HEK293 cells, SD‐208 markedly reduced sEV‐associated CD63 (Figure 7F,G) and induced substantial intracellular CD63 accumulation (Figure 7H), with densitometry showing an approximately 2.5‐fold increase compared to controls (Figure 7J). SD‐208 also significantly upregulated LAMP1, LC3A/B, and TOM20 (Figure 7I,J), with fold changes comparable to those observed in HEK293 cells. The consistency of these molecular alterations across two distinct cell types suggests a potentially conserved mechanism by which SD‐208 disrupts vesicle trafficking.

To determine whether SD‐208 altered specific tetraspanin‐positive EV populations, we next examined CD63, CD9, and CD81 in whole‐cell lysates and in EV fractions normalised either to equal particle number or to equivalent cell number. SD‐208 increased intracellular CD81 and CD9 modestly (Figure S12A–C), consistent with the previously observed rise in CD63. When EVs were loaded at equal particle number, CD63, CD9, and CD81 were all increased in the SD‐208 condition, indicating altered EV composition (Figure S12D–G). However, when EVs were normalised to cell number, CD9 and CD81 were reduced, consistent with the overall decrease in EV secretion (Figure S12H–J).

Confocal immunofluorescence microscopy with organellespecific markers helped determine the subcellular fate of accumulated CD63+ vesicles. In HEK293 cells, control conditions showed diffuse cytoplasmic CD63 staining with some punctate structures (Figure S13A), while SD‐208 treatment induced dramatic reorganization with prominent accumulation of CD63 in large vesicular structures throughout the cytoplasm. TOM20 staining revealed an expanded mitochondrial network (Figure S13B), consistent with western blot data and suggestive of altered mitochondrial homeostasis.

Co‐staining of CD63 and LC3A/B showed increased LC3 puncta following SD‐208 treatment, indicative of autophagosome accumulation or impaired turnover (Figure S13C). However, CD63 and LC3A/B displayed minimal spatial overlap; merged images revealed primarily adjacent rather than colocalized structures. Thus, although autophagy is activated, CD63+ vesicles are not predominantly trafficked through autophagic degradation.

In contrast, CD63 and LAMP1 showed extensive colocalization in SD‐208‐treated HEK293 cells (Figure S13D). High‐magnification views revealed clear co‐residence of both markers within the same vesicular structures, indicating robust targeting of CD63+ MVBs to lysosomes. Cardiac fibroblasts exhibited highly similar patterns (Figure S14): diffuse CD63 under control conditions, SD‐208‐induced punctate accumulation, increased TOM20 signal, minimal CD63–LC3A/B colocalization, and extensive CD63–LAMP1 colocalization (Figure S14A–D). These consistent phenotypes reinforce lysosomal redirection as the dominant fate of CD63+ vesicles.

**FIGURE 8:**
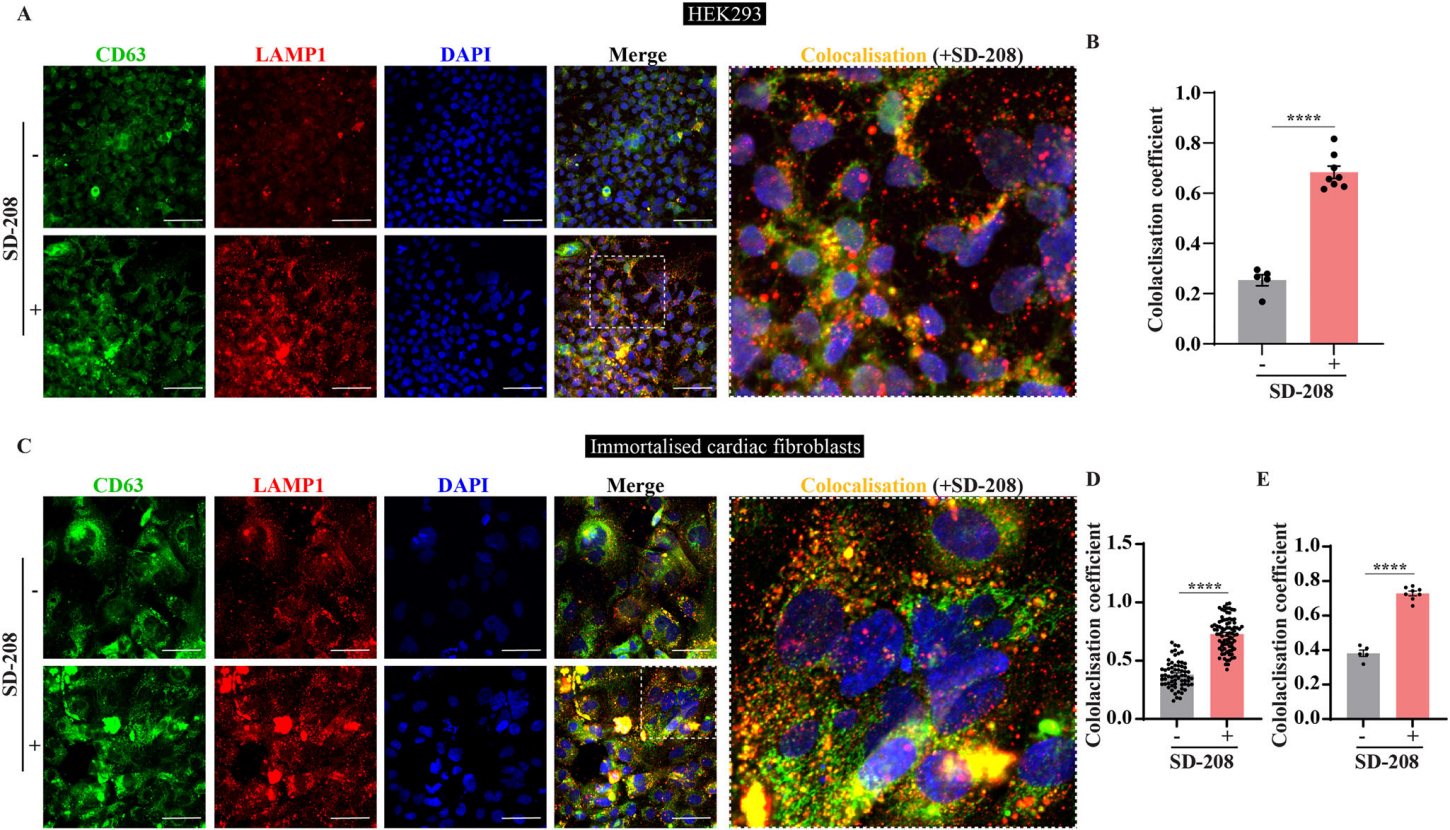
Quantitative colocalization analysis confirms preferential targeting of CD63+ vesicles to lysosomal compartments. (A, B) CD63‐LAMP1 colocalization in HEK293 cells. (A) Representative high‐resolution confocal images showing CD63 (green), LAMP1 (red), DAPI (blue), merged channels, and colocalization (yellow indicates overlap). Enlarged inset demonstrates extensive CD63‐LAMP1 colocalization in SD‐208‐treated cells. (B) Quantification of Manders' colocalization coefficients in control versus SD‐208‐treated (3 µM, 96 h) cells. Each data point represents one image containing >50 cells. (C‐E) CD63‐LAMP1 colocalization in immortalised cardiac fibroblasts. (C) Representative confocal images showing CD63 (green), LAMP1 (red), DAPI (blue), merged channels, and colocalization panel with enlarged inset. (D) Single‐cell quantification of CD63‐LAMP1 Manders' colocalization coefficients. Each data point represents one individual cell. (E) Image‐based quantification where each data point represents one image containing >15 cells. Scale bars; 50 µm (main images). Data represent mean ± SEM, *n* = 3 independent biological replicates. ****p < 0.0001 by unpaired *t*‐test.

High‐resolution image quantification showed minimal CD63–LAMP1 colocalization in control HEK293 cells (coefficient ∼0.2), which increased more than three‐fold upon SD‐208 treatment (∼0.65; Figure 8A,B). Each data point reflects an independent image containing >50 cells, demonstrating robust reproducibility. Cardiac fibroblasts showed similar increases in CD63–LAMP1 colocalization at both single‐cell and field‐of‐view levels (Figure 8C–E), with each image containing >15 cells. These analyses confirm that lysosomal routing is a consistent and dominant mechanism in both cell types.

Together, these findings support a model in which SD‐208 inhibits sEV secretion by affecting vesicle trafficking, without apparent impairment of MVB biogenesis. Intracellular accumulation of CD63, combined with its depletion from sEVs, indicates that MVBs form normally but fail to fuse with the plasma membrane. Instead, SD‐208 selectively redirects CD63+ MVBs to LAMP1+ lysosomal compartments, with minimal engagement of LC3A/B+ autophagosomes despite elevated LC3 levels. This represents a targeted disruption of the secretory arm of the endosomal system while engaging compensatory lysosomal pathways to handle the accumulated vesicular cargo. The upregulation of TOM20 and expansion of mitochondrial networks may reflect secondary adaptations to altered cellular metabolism or disrupted organellar quality control mechanisms. Importantly, the remarkable consistency of these mechanistic features across HEK293 cells and cardiac fibroblasts—two cell types with vastly different biological contexts—demonstrates that SD‐208 targets a conserved and fundamental aspect of vesicle trafficking machinery that is independent of cell type or activation state.

## 4 Discussion

In this study, we uncover a previously unrecognised function of the selective ALK5 inhibitor SD‐208 as a potent small‐molecule inhibitor of small extracellular vesicle (sEV) secretion. Using primary cardiac fibroblasts from HCM patients, immortalised cardiac fibroblasts, and non‐fibrotic HEK293 cells, we demonstrate that SD‐208 reduces EV release independently of its anti‐fibrotic actions. Mechanistic analyses revealed that SD‐208 disrupts vesicle trafficking rather than biogenesis, diverting CD63^+^ MVBs from secretion toward lysosomal degradation. These findings establish SD‐208 may serve as a pharmacological tool to interrogate EV trafficking and as a potential therapeutic candidate to modulate EV‐driven pathology.

**FIGURE 9:**
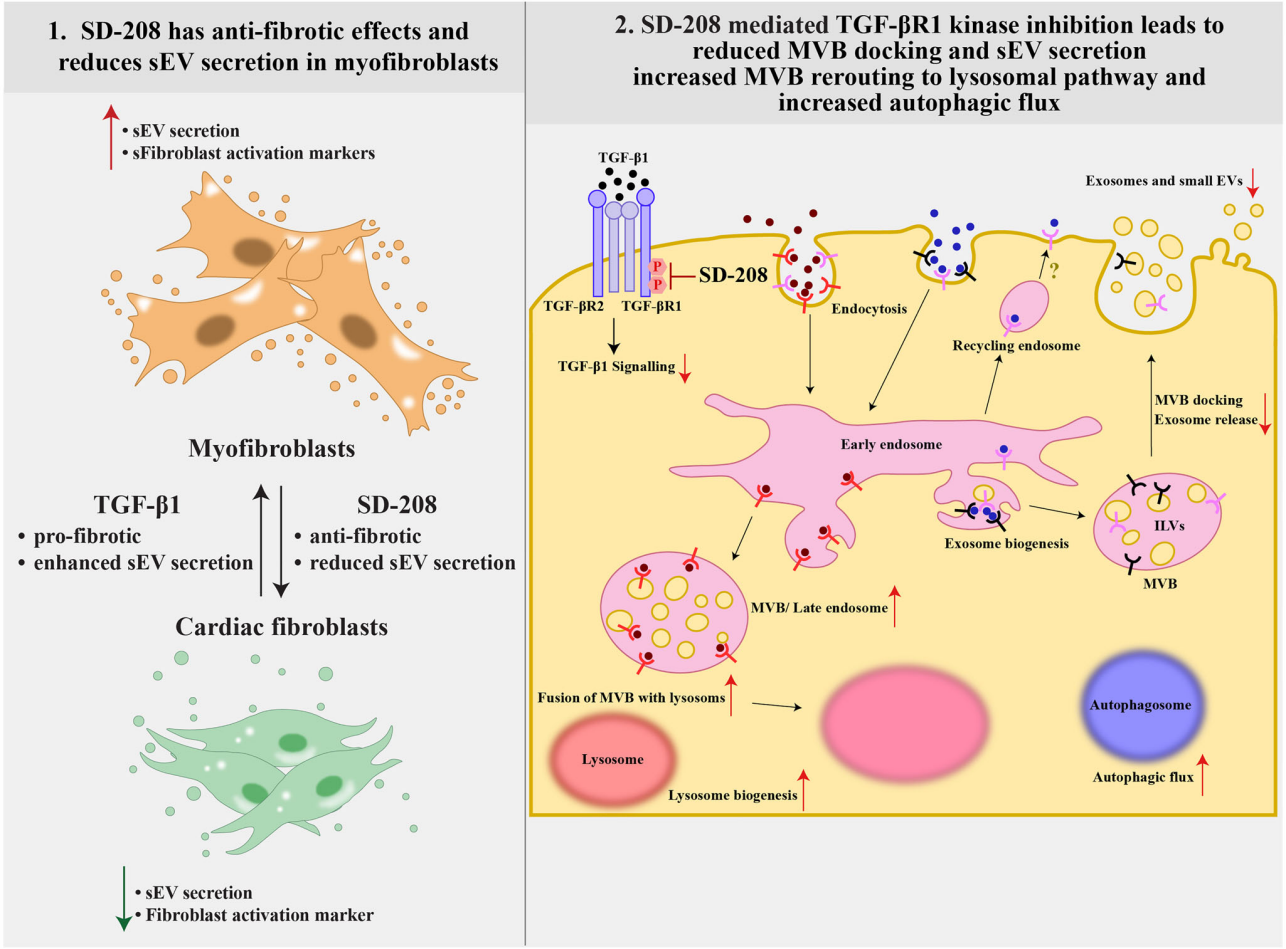
Proposed model of SD‐208‐mediated regulation of fibroblast activation and extracellular vesicle trafficking. (1) SD‐208 exerts anti‐fibrotic effects through inhibition of TGF‐β receptor I (ALK5) signalling while also reducing sEV secretion in activated myofibroblasts. TGF‐β1 promotes fibroblast activation and enhances sEV secretion, whereas SD‐208 suppresses both processes. (2) SD‐208 impairs MVB docking at the plasma membrane, reducing exocytosis and sEV release. Retained MVBs are redirected to lysosomes for degradation, with enhanced lysosome biogenesis (LAMP1↑) and dysregulated autophagic flux (LC3A/B↑). ILVs, intraluminal vesicles; MVB, multivesicular body.

We propose a dual mechanism of action for SD‐208 (Figure 9): in activated myofibroblasts, it exerts canonical anti‐fibrotic effects through ALK5 inhibition while simultaneously reducing sEV secretion; in non‐activated cells, it independently disrupts vesicle trafficking machinery, diverting MVBs from plasma membrane fusion toward lysosomal degradation. This model may explain SD‐208's ability to reduce EV secretion across diverse cell types in vitro, regardless of activation state.

### 4.1 SD‐208 as a Small‐Molecule Regulator of EV Secretion

Pharmacological modulation of EV secretion remains a major unmet need in extracellular vesicle research (Catalano and O'Driscoll 2020; van Niel et al. 2018; Colombo et al. 2014). Although genetic strategies targeting Rab GTPases or ESCRT components have advanced mechanistic understanding, they are not clinically tractable (Catalano and O'Driscoll 2020; Colombo et al. 2013; Hsu et al. 2010; Ostrowski et al. 2010). Existing small‐molecule inhibitors such as GW4869, manumycin A, and calpeptin act indirectly, often affecting cell viability, lipid metabolism, or signalling cascades, thereby confounding interpretation of their effects on EV biology (Catalano and O'Driscoll 2020; Li et al. 2022; Datta et al. 2017; Menck et al. 2017; Panigrahi et al. 2018). Moreover, inhibition of specific EV biogenesis pathways may induce compensatory activation of alternative secretion mechanisms. For example, GW4869, a neutral sphingomyelinase inhibitor that reduces ceramide‐dependent exosome release, has been reported in certain contexts to alter vesicle output dynamics, including increased shedding of plasma membrane–derived ectosomes, underscoring the plasticity of vesicle trafficking networks and the complexity of pharmacologically targeting EV pathways (Catalano and O'Driscoll 2020; Jacquemyn et al. 2026).

In contrast, our findings position SD‐208 as a distinctive addition to this limited toolkit. SD‐208 reproducibly reduces small EV secretion across multiple cell types without detectable impairment of cell viability, proliferation, or metabolic activity. Importantly, we did not observe shifts in particle size distribution suggestive of increased release of larger vesicle populations under our experimental conditions. Together, these observations suggest that SD‐208 modulates vesicle trafficking fate rather than simply inhibiting a single biogenesis pathway, highlighting its potential utility as both an experimental probe to dissect endosomal routing mechanisms and a starting point for the development of EV‐targeted therapeutic strategies. Although our data support enhanced lysosomal routing of CD63^+^ compartments following SD‐208 treatment, we did not directly quantify potential changes in other EV subpopulations, such as larger ectosomes. Future studies using orthogonal isolation and characterization approaches will be required to determine whether compensatory shifts in vesicle subtypes occur.

### 4.2 Mechanistic Insights: Lysosomal Rerouting of MVBs

We show that SD‐208 treatment causes intracellular accumulation of CD63, increased colocalization with the lysosomal marker LAMP1, and depletion of CD63 in secreted EVs. These observations indicate that SD‐208 alters the fate of MVBs, promoting their fusion with lysosomes for degradation rather than with the plasma membrane for vesicle release. In essence, SD‐208 shifts the trafficking equilibrium away from the secretory MVB–plasma membrane route toward the degradative MVB–lysosome pathway, thereby reducing EV secretion without affecting vesicle formation itself.

This redirection of MVBs likely reflects impaired docking or fusion competency at the plasma membrane, potentially through disruption of Rab GTPase‐dependent exocytic machinery or altered calcium‐dependent fusion kinetics (Hsu et al. 2010; Ostrowski et al. 2010; Im et al. 2019; Savina et al. 2005). Proteomic profiling supports this mechanism, revealing intracellular accumulation of proteins involved in endosomal trafficking and vesicle secretion machinery alongside enrichment of late endosome markers, ubiquitin ligase complex components, and proteins associated with endosome‐to‐lysosome transport and autophagy (including ATG4D). Cellular component analysis demonstrated intracellular accumulation of extracellular exosome, extracellular vesicle, and endocytic compartment proteins, consistent with vesicle retention rather than secretion. The simultaneous enrichment of mitochondrial and endoplasmic reticulum proteins suggests a broader perturbation of cellular membrane dynamics and endosomal–lysosomal homeostasis, potentially reflecting compensatory responses to disrupted vesicle trafficking (Song et al. 2019; Underwood et al. 2020; Liang et al. 2023). A comprehensive summary of these proteomic changes and key differentially expressed proteins identified by western blot and confocal microscopy is provided in Table S6. Subsequent validation by western blot confirmed protein‐level increases in the lysosomal marker LAMP1, autophagosome marker LC3A/B, and mitochondrial marker TOM20, while confocal microscopy revealed that accumulated CD63+ MVBs preferentially colocalise with LAMP1+ lysosomes rather than LC3A/B+ autophagosomes. The marked elevation of LC3A/B, coupled with the preferential CD63‐LAMP1 colocalization, suggests that while autophagic pathways are engaged, the primary route of MVB disposal is through direct lysosomal fusion rather than autophagosome‐mediated degradation.

This process aligns with emerging evidence that the balance between MVB–plasma membrane and MVB–lysosome fusion is a critical checkpoint controlling EV secretion. Perturbations in calcium signalling, endosomal pH, membrane lipid composition, or protein ubiquitination status can alter this balance (Savina et al. 2005; Villarroya‐Beltri et al. 2016; Sung et al. 2020; Cen et al. 2022; Abdullah et al. 2021). The strong enrichment of calcium ion transmembrane transport and ubiquitin‐dependent protein catabolic pathways in our proteomic data suggests that SD‐208 may influence one or more of these regulatory nodes. The substantial upregulation of ubiquitin ligase complexes (300‐fold enrichment) is particularly striking and may indicate enhanced protein ubiquitination affecting vesicle trafficking machinery or altered sorting of ubiquitinated cargo within MVBs (Villarroya‐Beltri et al. 2016; Cen et al. 2022; Farooq et al. 2022). Further work using live‐cell trafficking assays, pH‐sensitive reporters, and real‐time imaging of MVB‐lysosome fusion events will help delineate how SD‐208 dynamically modulates these processes.

### 4.3 Independence From Fibrotic Signalling

A key finding of this study is that SD‐208 significantly reduced EV secretion even in non‐activated cardiac fibroblasts and non‐fibrotic HEK293 cells lacking active TGF‐β/Smad signalling (Stepanenko and Dmitrenko 2015; Liu et al. 2016). In ICF, SD‐208 produced minimal changes in myofibroblast activation markers yet substantially reduced EV secretion, while in HEK293 cells—which lack the fibrotic machinery entirely—SD‐208 produced comparable reductions in vesicle release. This dissociation clearly demonstrates that SD‐208's vesicle‐modulatory action is independent of its canonical ALK5‐mediated anti‐fibrotic effects and instead represents a conserved effect on fundamental vesicle trafficking machinery.

Although developed as a selective TGF‐β receptor I (ALK5) kinase inhibitor, SD‐208 may influence additional kinase networks involved in endosomal trafficking, vesicle sorting, or membrane fusion. Off‐target inhibition of kinases regulating vesicle trafficking pathways, including those associated with Rab GTPase function, SNARE protein function, and calcium‐dependent exocytosis could partly account for the observed effects. Indeed, kinases regulating Rab GTPase activity, SNARE‐mediated fusion, and calcium‐dependent exocytosis are known to be modulated by ATP‐competitive inhibitors in other contexts (Barclay et al. 2005; Seol et al. 2019; Levin et al. 2016; Belluzzi et al. 2016). Although direct overlap with TGF‐βRI inhibitors like SD‐208 has not been conclusively demonstrated, these pathways provide plausible off‐target or parallel mechanisms that could affect EV secretion. Comprehensive kinome profiling and phosphoproteomic analysis will be essential to identify non‐canonical targets underlying SD‐208's EV‐inhibitory activity and to determine whether this effect is unique to SD‐208 or shared among structurally related ALK5 inhibitors.

Although Rab GTPases are key regulators of vesicle trafficking and EV secretion, our proteomic analyses in both HEK293 cells and primary cardiac myofibroblasts did not reveal a consistent global decrease in Rab family proteins following SD‐208 treatment (Figure S15, Tables S7 and S8). Instead, Rab protein levels showed heterogeneous or modest changes, with no coordinated pattern indicative of Rab‐driven reduction of EV secretion. These findings suggest that SD‐208 does not act through broad alterations in Rab protein abundance, but rather through modulation of downstream vesicle trafficking pathways, consistent with the enhanced lysosomal routing of CD63^+^ compartments observed in this study.

Consistent with the broader proteomic findings, STRING network analysis of proteins downregulated following SD‐208 treatment in HEK293 cells revealed coordinated suppression of receptor signalling, WNT/developmental pathways, vesicle trafficking and membrane transport, cytoskeletal organisation, cilia‐associated processes, and ubiquitin‐mediated protein turnover (Figure S16). These network‐level changes further support the concept that SD‐208 exerts pleiotropic effects on cellular trafficking and signalling pathways rather than acting solely through canonical ALK5‐mediated transcriptional responses.

### 4.4 Implications for Disease and Therapy

EVs are central mediators of intercellular communication in diverse pathologies, including fibrosis, cancer, neurodegeneration, and cardiovascular disease. In the heart, fibroblast‐derived EVs contribute to cardiomyocyte hypertrophy, inflammation, and electrical remodelling—key features of HCM and other fibrotic cardiovascular conditions (Bang et al. 2014; Wang et al. 2022; Sanwlani et al. 2025; Sanwlani and Camelliti 2026; Thompson et al. 2024; Tian et al. 2020; Lyu et al. 2015). The ability of SD‐208 to both attenuate fibroblast activation and inhibit EV secretion suggests a dual therapeutic potential: limiting both direct fibrotic signalling through canonical TGF‐β pathways and reducing vesicle‐mediated propagation of pathological cues to cardiomyocytes and immune cells (Ranjan et al. 2021; Akbar et al. 2023; Johnson et al. 2023; Fujioka et al. 2023). Importantly, SD‐208 did not completely abolish sEV secretion, but rather produced a consistent and significant reduction in vesicle output across all cell types examined. This combined mechanism may provide synergistic benefit in treating cardiac fibrosis and remodelling diseases where both cell‐intrinsic activation and paracrine EV signalling contribute to pathology. Beyond the cardiovascular system, this mechanism could be relevant in cancer, where tumour‐derived EVs promote metastasis, angiogenesis, and immune evasion, and in neurodegenerative diseases, where EVs propagate toxic protein aggregates (Costa‐Silva et al. 2015; Guo et al. 2015; Fonseka et al. 2021). The ability to pharmacologically suppress EV secretion without compromising cellular viability or metabolism makes SD‐208 a promising candidate for further therapeutic development in any disease context where modulating vesicle release could provide clinical benefit. Moreover, the identification of lysosomal rerouting as the mechanism of action aligns with recent findings linking endosomal–lysosomal cross‐talk to vesicle secretion (Im et al. 2019; Sahu et al. 2011; McAndrews et al. 2019) and suggests that combination strategies targeting both EV release and lysosomal degradation capacity might further enhance therapeutic efficacy.

### 4.5 Limitations and Future Directions

Although our findings clearly demonstrate that SD‐208 inhibits EV secretion and alters vesicle trafficking through lysosomal rerouting, several important questions remain. First, the direct molecular targets responsible for disrupted vesicle trafficking require further investigation. Although the current study provides in‐depth mechanistic context, it does not identify a single molecular target. This reinforces the need for future kinome profiling, phosphoproteomic studies and chemical biology approaches using modified SD‐208 analogues to identify the kinase activities essential for the EV‐inhibitory effect and define the direct pathways underlying the EV‐suppressive phenotype. Second, the absence of in vivo validation is a limitation that must be addressed; assessing whether systemic SD‐208 administration alters circulating EV levels, tissue EV content, or cardiac remodelling in HCM or heart failure models will be critical next steps. Third, expanding the analysis to other ALK5 inhibitors (such as SB‐431542, LY‐364947, or galunisertib) or chemical analogues may clarify whether this EV‐modulatory effect is unique to SD‐208's specific chemical structure or represents a broader class effect of ALK5 inhibition (Teixeira et al. 2025; Xie et al. 2022; Peterson et al. 2022). Notably, SB431542 has been reported to increase EV production in MS‐5 stromal cells, indicating that ALK5 inhibition can alter EV output in a context‐dependent manner (Gautheron et al. 2023). In line with this, our data (Figure S7E–H) show that neither SB‐431542 nor galunisertib phenocopied the SD‐208‐mediated reduction in EV secretion in HEK293 cells, supporting the conclusion that this phenotype is compound‐ and context‐dependent rather than a uniform class effect of ALK5 inhibition.

Additionally, understanding the temporal dynamics of MVB rerouting—specifically, whether this is a rapid effect on pre‐existing trafficking machinery or requires longer‐term transcriptional changes—will inform optimal dosing strategies. Finally, investigating whether SD‐208's effects are reversible and whether prolonged EV suppression has unintended consequences on intercellular communication in physiological contexts will be important for therapeutic development. The substantial mitochondrial changes observed suggest that long‐term treatment effects on cellular energetics and organellar quality control should also be carefully monitored.

## 5 Conclusions

Our findings identify SD‐208 as a dual‐function small molecule that inhibits fibroblast activation while independently suppressing extracellular vesicle secretion through lysosomal rerouting of CD63^+^ MVBs. This discovery establishes SD‐208 as both a pharmacological tool to dissect vesicle trafficking checkpoints and a potential therapeutic strategy to limit EV‐mediated intercellular communication in disease. By uncovering a previously unrecognized trafficking mechanism that shifts MVB fate from plasma membrane fusion to lysosomal degradation, this work bridges EV biology, fibrosis, and pharmacology, laying a foundation for the development of clinically relevant modulators of vesicle secretion. The independence of this effect from canonical TGF‐β signalling, combined with its conservation across diverse cell types, suggests that SD‐208 targets fundamental aspects of endosomal trafficking that may be therapeutically exploitable across multiple disease contexts where pathological EV secretion drives disease progression.

## Author Contributions

Made substantial contributions to conception and design of the study and performed data curation, analysis and interpretation: Rahul Sanwlani and Patrizia Camelliti. Funding acquisition: Patrizia Camelliti, John H. McVey, Konstantinos Savvatis, Aled Clayton. Performed data acquisition: Rahul Sanwlani, Georgina H. Thompson, Kyle Bramich, Ching‐Seng Ang, Vlad Stolojan, Aled Clayton, Patrizia Camelliti. Provided administrative, technical, and material support: Ching‐Seng Ang, Millie L. Trowbridge, Chris E. Duringer, Konstantinos Savvatis, Aled Clayton, Suresh Mathivanan, Sean M. Davidson, John H. McVey, Patrizia Camelliti. The original manuscript was drafted by Rahul Sanwlani, and figures were prepared by Rahul Sanwlani and Georgina H. Thompson. Final manuscript was edited and reviewed by Rahul Sanwlani and Patrizia Camelliti. All authors commented on previous versions of the manuscript. All authors read and approved the final manuscript.

## Funding

Rahul Sanwlani and Patrizia Camelliti are funded by the British Heart Foundation Project Grant PG/22/11172. The authors also acknowledge support from the Royal Society (RSG/R1/180198), the John Jacob Astor Charitable Trust and the Inman Charity. The funders had no role in study design, data collection and analysis, decision to publish, or preparation of the manuscript.

## Consent

All authors consent to the publication of this manuscript.

## Conflicts of Interest

The authors declare no conflicts of interest.

## Supporting information




**Supporting Information**: jev270362‐sup‐0001‐TableS1.xlsx

**Supporting Information**: jev270362‐sup‐0002‐TableS2‐S4.docx

**Supporting Information**: jev270362‐sup‐0003‐TableS5.xlsx

**Supporting Information**: jev270362‐sup‐0004‐TableS6.xlsx

**Supporting Information**: jev270362‐sup‐0005‐TableS7‐S8.xlsx

**Supporting Information**: jev270362‐sup‐0006‐TableS9.xlsx

**Supporting Information**: jev270362‐sup‐0007‐TableS10‐17.xlsx

**Supporting Information**: jev270362‐sup‐0008‐Figure S1.jpg

**Supporting Information**: jev270362‐sup‐0009‐FigureS2.jpg

**Supporting Information**: jev270362‐sup‐0010‐FigureS3.jpg

**Supporting Information**: jev270362‐sup‐0011‐FigureS4.jpg

**Supporting Information**: jev270362‐sup‐0012‐FigureS5.jpg

**Supporting Information**: jev270362‐sup‐0013‐FigureS6.jpg

**Supporting Information**: jev270362‐sup‐0014‐FigureS7.jpg

**Supporting Information**: jev270362‐sup‐0015‐FigureS8.jpg

**Supporting Information**: jev270362‐sup‐0016‐FigureS9.jpg

**Supporting Information**: jev270362‐sup‐0017‐FigureS10.jpg

**Supporting Information**: jev270362‐sup‐0018‐FigureS11.jpg

**Supporting Information**: jev270362‐sup‐0019‐FigureS12.jpg

**Supporting Information**: jev270362‐sup‐0020‐FigureS13.jpg

**Supporting Information**: jev270362‐sup‐0021‐FigureS14.jpg

**Supporting Information**: jev270362‐sup‐0022‐FigureS15.jpg

**Supporting Information**: jev270362‐sup‐0023‐FigureS16.jpg

## Data Availability

Proteomics data are available within the supplementary material. The datasets generated and analysed during the current study are available from the corresponding author on reasonable request.
