# Supplementary material for: Pharmacological Inhibition of Small Extracellular Vesicle Secretion by ALK5i SD‐208 via Lysosomal Rerouting of CD63+ Compartments
Source: J Extracell Vesicles. 2026 Aug 29;15(9):e70362. doi: 10.1002/jev2.70362 (PMC13525873; doi:10.1002/jev2.70362)
Supplement: Supplementary file 1 [file JEV2-15-e70362-s019.docx]

**Supplementary table 2-** **List of primary antibodies, isotype controls and secondary antibodies used in immunofluorescence (IF) and flow cytometry (F)**

| **Antibody** | **Species** | **Dilution** | **Source** | **Application** | **Reference** |
| --- | --- | --- | --- | --- | --- |
| α-SMA | Mouse anti-human monoclonal | 1:100 | DAKO | IF, F | M0851 |
| CD31 | Rabbit anti-human, polyclonal | 1:200 | Abcam | IF | ab28364 |
| Vimentin | Mouse anti-human | 1:1000 | Invitrogen | IF | A1-06908 |
| LAMP1 D2D11 XP(R) | Rabbit anti-human monoclonal | 1:200 | Cell Signalling Technology | IF | 9091S |
| LC3A/B D 3U4C XP(R) | Rabbit anti-human monoclonal | 1:200 | Cell Signalling Technology | IF | 12741S |
| TOM20 F-10 | Mouse anti-human monoclonal | 1:200 | Santa Cruz Biotechnology | IF | sc-365062 |
| CD63 (TS63) | Mouse anti-human monoclonal | 1:200 | Invitrogen | IF | 10628D |
| CD90 | Mouse anti-human monoclonal | 1:20 | BioLegend | F | 328121 |
| CD90 isotype | Mouse anti-human monoclonal | 1:20 | BioLegend | F | 400157 |
| CD31 | Mouse anti-human monoclonal | 1:20 | BioLegend | F | 303119 |
| CD31 isotype | Mouse anti-human monoclonal | 1:20 | BioLegend | F | 400127 |
| CD45 | Mouse anti-human monoclonal | 1:20 | BioLegend | F | 368513 |
| CD45 isotype | Mouse anti-human monoclonal | 1:20 | BioLegend | F | 400142 |
| Fibronectin | Rabbit anti-human monoclonal | 1:200 | Abcam | IP, F | AB2413 |
| Ki-67 | Rabbit anti-human monoclonal | 1:200 | Proteintech | IF | AB9260 |
| Alexa Fluor 488 | Donkey anti-mouse monoclonal | 1:200 | Invitrogen | IF, F | A-21202 |
| Alexa Fluor 488 | Goat anti-rabbit, polyclonal | 1:200 | Invitrogen | IF | A-11008 |

**Supplementary table 3- List of primary antibodies used for western blotting**

| **Antibody** | **Species** | **Dilution** | **Source** | **Reference** |
| --- | --- | --- | --- | --- |
| LAMP1 D2D11 XP(R) | Rabbit mAb | 1:1000 | Cell Signalling Technology | 9091S |
| LC3A/B D 3U4C XP(R) | Rabbit mAb | 1:1000 | Cell Signalling Technology | 12741S |
| GAPDH | Rabbit polyclonal | 1:1000 | Abcam | AB37168 |
| GAPDH | Mouse mAb | 1:1000 | Santa Cruz Biotechnology | sc-365062 |
| TOM20 F-10 | Mouse mAb | 1:1000 | Santa Cruz Biotechnology | sc-17764 |
| CD63 (TS63) | Mouse mAb | 1:1000 | Invitrogen | 10628D |
| α-SMA | Mouse mAb | 1:1000 | DAKO | M0851 |
| COL1A, COL3A1 | Rabbit polyclonal | 1:1000 | Abcam | AB34710 |
| Alix | Rabbit polyclonal | 1:1000 | Abcam | Ab88388 |
| TSG101 | Mouse mAB | 1:1000 | GeneTex | GTX70255 |
| Syntenin-1 | Rabbit mAB | 1:1000 | Cell Signalling Technology | 27964 |
| Flotillin-1 | Mouse mAB | 1:1000 | BD Biosciences | 610820 |
| Calnexin | Rabbit polyclonal | 1:1000 | Abcam | Ab22595 |

**Supplementary table 4- List of secondary antibodies used for western blotting**

| **Antibody** | **Species** | **Dilution** | **Source** | **Reference** |
| --- | --- | --- | --- | --- |
| IRDye® 680 RD | Goat anti-Rabbit | 1:20,000 | LI-COR | 926-68071 |
| IRDye® 680 RD | Goat anti-mouse | 1:20,000 | LI-COR | 926-68070 |
| IRDye® 800 CW | Goat anti-Rabbit | 1:20,000 | LI-COR | 926-33211 |
| IRDye® 800 CW | Goat anti-mouse | 1:20,000 | LI-COR | 926-32210 |
